# Immune Response in Severe and Non-Severe Coronavirus Disease 2019 (COVID-19) Infection: A Mechanistic Landscape

**DOI:** 10.3389/fimmu.2021.738073

**Published:** 2021-10-13

**Authors:** Kavitha Mukund, Priya Nayak, Chethan Ashokkumar, Sohail Rao, Jose Almeda, Monica M. Betancourt-Garcia, Rakesh Sindhi, Shankar Subramaniam

**Affiliations:** ^1^ Department of Bioengineering, University of California, San Diego, La Jolla, CA, United States; ^2^ Plexision Inc., Pittsburgh, PA, United States; ^3^ Hillman Center for Pediatric Transplantation, University of Pittsburgh, Pittsburgh, PA, United States; ^4^ DHR Health and DHR Health Institute for Research and Development, Edinburg, TX, United States; ^5^ Department of Cellular and Molecular Medicine, University of California, San Diego, La Jolla, CA, United States; ^6^ Department of Computer Science and Engineering, University of California, San Diego, La Jolla, CA, United States

**Keywords:** COVID-19, MDSC, immune-remodeling, plasmablast, low-density neutrophils, megakaryocyte, aggregation, cDC2

## Abstract

The mechanisms underlying the immune remodeling and severity response in coronavirus disease 2019 (COVID-19) are yet to be fully elucidated. Our comprehensive integrative analyses of single-cell RNA sequencing (scRNAseq) data from four published studies, in patients with mild/moderate and severe infections, indicate a robust expansion and mobilization of the innate immune response and highlight mechanisms by which low-density neutrophils and megakaryocytes play a crucial role in the cross talk between lymphoid and myeloid lineages. We also document a marked reduction of several lymphoid cell types, particularly natural killer cells, mucosal-associated invariant T (MAIT) cells, and gamma-delta T (γδT) cells, and a robust expansion and extensive heterogeneity within plasmablasts, especially in severe COVID-19 patients. We confirm the changes in cellular abundances for certain immune cell types within a new patient cohort. While the cellular heterogeneity in COVID-19 extends across cells in both lineages, we consistently observe certain subsets respond more potently to interferon type I (IFN-I) and display increased cellular abundances across the spectrum of severity, as compared with healthy subjects. However, we identify these expanded subsets to have a more muted response to IFN-I within severe disease compared to non-severe disease. Our analyses further highlight an increased aggregation potential of the myeloid subsets, particularly monocytes, in COVID-19. Finally, we provide detailed mechanistic insights into the interaction between lymphoid and myeloid lineages, which contributes to the multisystemic phenotype of COVID-19, distinguishing severe from non-severe responses.

## 1 Introduction

On track to becoming endemic, the severe acute respiratory syndrome coronavirus 2 (SARS-CoV-2) virus has triggered the global pandemic of coronavirus disease 2019 (COVID-19), a complex infection with multisystemic effects (1). Most respiratory viruses such as the other beta-coronaviruses, respiratory syncytial virus (RSV), influenza, and rhinovirus, trigger a potent innate and adaptive immune response leading to a rapid resolution of infection and generation of circulating memory cells, which can combat a reinfection (2–4). Current research however has indicated a more heterogeneous response to SARS-CoV-2 in humans, with symptoms ranging from mild to severe, resulting in hospitalization and mortality (4–7) in some. Severe COVID-19 involves an extensive cross talk between the activated immune system and other physiological mechanisms, in many cases leading to multisystem comorbidities including acute respiratory distress, septic shock, seizure, renal failure, heart attack, and thromboembolism (8). Recent research has extensively utilized high-throughput techniques such as single-cell RNA sequencing (scRNAseq) and high-throughput flow cytometry techniques to catalogue the immune cell-state changes contributing to SARS-CoV-2 response [e.g., (5, 7, 9–11)]. Broadly, these studies have identified a dramatic remodeling of the major immune players. Specifically, monocytes and neutrophils (and their several novel subtypes) have been described to undergo expansion correlating with disease severity in COVID-19 subjects. COVID-19 research has also focused largely on deciphering the role of adaptive immunity, particularly CD4T, CD8T, and B cells (naïve and mature) in severe, non-severe, and convalescent/recovered COVID-19 [e.g., (10, 12–19)]. These studies and many others have identified impaired activation and reduced cytotoxicity arising from the adaptive arm including B and CD8T cells within severe disease. A higher percentage of activated and proliferative CD8T population has been documented within less severe infections (19). Despite reduced CD4T frequencies, these studies have suggested normal activity for CD4T within COVID-19 (12). T cells from COVID-19 patients have shown significantly higher levels of exhaustion with increasing severity (14). Research has shown that though the plasma B and proliferative T-cell repertories correlate with severity, compositional differences of their precursors are influenced heavily by age and sex (16) and exhibit reasonably robust long-term memory against SARS-CoV-2 (18).

Several of these studies, however, have been limited by cohort sizes, leading to interpretations that while contextually correct may be non-comprehensive. More importantly, these studies do not provide an integrative mechanistic understanding of the remodeling of the immune system and the concomitant alterations in immune response. To overcome this limitation, we analyzed scRNAseq data from four published studies from peripheral blood mononuclear cells (PBMCs) of COVID-19 patients: Lee et al. (4), Wilk et al. (10), Schulte-Schrepping et al. (5), and Arunachalam et al. (20). These data together comprise a substantial cohort size (111 patients across ~350K cells) across 20 immune cell compartments of both lymphoid and myeloid lineage. Despite the larger cohort size, a simple linear analysis such as principal component analysis (PCA) on donors shows a heterogeneity among patient responses with no clean separation between non-severe and severe patients (results not shown). Such lower dimensional analyses demonstrate the need for more complex dimensionality reduction methods for delineating the differences between healthy, non-severe, and severe subjects, as demonstrated by our work. Our integrated analyses not only enhances the statistical power to enable functional and mechanistic insights into COVID-19, but allows for detection of low-frequency or transient cell types that may otherwise be poorly or superficially characterized in smaller cohorts. In contrast to two recently published integrated studies (16, 21), we focus largely on these less characterized immune cell subsets, which have either low frequencies or are not well explored within COVID-19 literature. Additionally, we identify a set of consensus gene signatures across severe and non-severe disease enabling the characterization of transcriptional signatures across all immune cell compartments. Taken together, our analyses allow us to put forth a mechanistic framework underlying the interconnected host immune responses, driving and distinguishing severe from non-severe immune responses within COVID-19.

## 2 Results

### 2.1 Integrating Single-Cell RNA Sequencing Data From COVID-19 Patients

**Figure 1:**
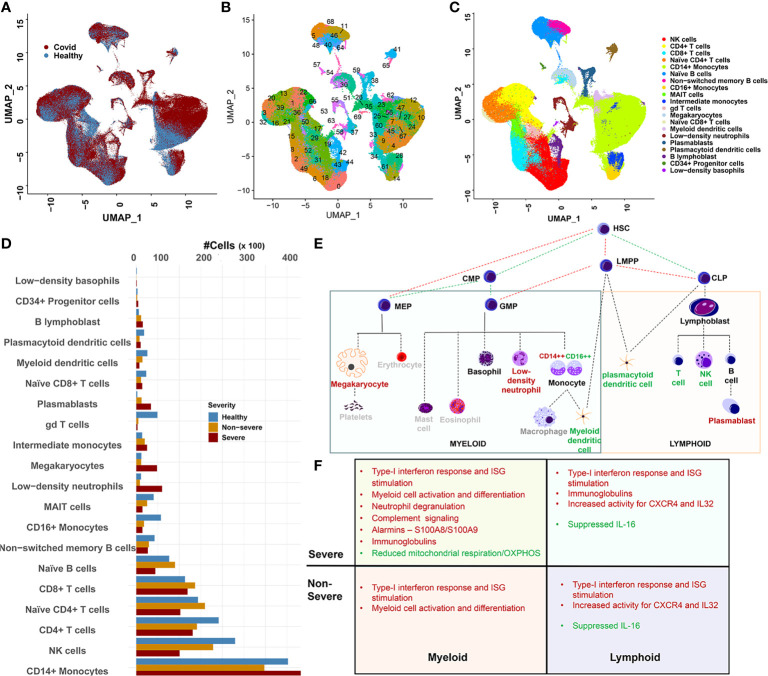
Overview of data integration and cellular heterogeneity. **(A)** UMAP embedding of the integrated dataset highlighting cell distribution from coronavirus disease 2019 (COVID-19) and healthy subjects. The integration was performed using Seurat 3.2 (see *Materials and Methods*) and represents the combined data from four studies including Arunachalam et al. (PA), Lee et al. (Lee), Schulte-Schrepping (two cohorts SS1 and SS2), and Wilk et al. (Wilk). **(B)** UMAP embedding of the 70 clusters detected within the integrated dataset. **(C)** Twenty distinct cellular compartments identified after grouping the 70 clusters based on automatic (SingleR) and manual annotation. **(D)** Abundance distribution of the 20 cell types across healthy, severe, and non-severe diseases. CD14^+^ monocytes showed the most drastic expansion within severe and non-severe diseases. Loss of cellular abundances associated within COVID-19 samples for mucosal-associated invariant T (MAIT) and gamma-delta T (γδT) was observed. **(E)** The downstream analysis of immune cell types was performed in the context of their origin. Red lettering indicates cell types that expand within COVID-19, green indicate cell types with reducing abundances in COVID-19, and gray indicates cell types unexplored within our current manuscript. HSC, hematopoietic stem cells; CLP, common lymphoid progenitor cells; LMPP, LYMPHOID-primed multi-potential progenitor cells; CMP, common myeloid progenitor cells; MEP, megakaryocyte–erythroid progenitor cells; GMP, granulocyte–macrophage progenitor cells. **(F)** Outline of the major functional features associated with the consensus gene signatures identified across lineages and severities.

Datasets for each of the published studies referenced above henceforth referred to as Lee (4), Wilk (10), SS_C1, SS_C2 [two cohorts from SS, (5)] and PA (20) were downloaded from Gene Expression Omnibus (GEO) or the sources as indicated in the original publications (see *Materials and Methods*). All donor relevant metadata information (from each study) used within our integrated dataset are provided in **Supplementary Tables S1–S3**. The original datasets were processed and integrated using Seurat v3.2 as outlined in the *Materials and Methods*, resulting in an integrated dataset of 375,438 single-cell transcriptomes from 111 donors (**Figure 1A** and **Supplementary Figures S1A, B**). The resulting parent Seurat object was scaled and clustered into 70 distinct clusters and visualized using UMAP embedding (**Figure 1B**). Grouping the 70 clusters based on their cellular identities revealed 20 distinct immune cell types (**Figure 1C** and **Supplementary Figure S1C**, see *Materials and Methods*). Monocytes were the largest major cell type identified, particularly CD14^+^ monocytes at ~31% (103,659 cells), with low-density basophils being the smallest population of recognizable cells. A visual inspection of the cellular frequencies across severities revealed some notable differences between the 20 compartments (**Figure 1D** and **Supplementary Figure S1D**). To discern the trends in cell-type changes and between cell-type interactions, we grouped the mononuclear cell types by origin into myeloid and lymphoid cell types (**Figure 1E**, see Section 4.6). Assuming that the cellular abundances correlated with levels of circulating cells, we observed significant gains (p < 0.05) in abundances within COVID-19 (compared with healthy) for monocytes and several low-frequency cell types including, low-density neutrophils (LDNs), megakaryocytes (MKs), and plasmablasts (PBs), consistent with extant research (5, 6, 21). A progressive loss of several other cell types including dendritic cells (DCs), γδT cells, natural killer (NK) cells, and mucosal-associated invariant T (MAIT) was also observed (**Figure 1E** and **Supplementary Figure S1D**).

### 2.2 Common Transcriptional Signatures Between Myeloid and Lymphoid Cell Types in COVID-19

We sought to first understand if a common program of transcriptional dysregulation exists across the myeloid and lymphoid milieu and if it differs across severities. Towards this, we extracted differentially expressed genes (DEGs) by comparing cells from severe and non-severe with healthy cells, independently, for each of the 20 immune cell compartments (see *Materials and Methods*). A consensus gene set was defined as DEGs that were present in at least 50% of all cell types contained within each lineage for severe and non-severe disease (**Supplementary Figure S2A, Supplementary Tables S5** and **S6**). An inspection of the consensus gene signatures across lineages and severities highlighted the following four broad features (**Figure 1F**). First, significant dysregulation of genes associated with interferon type I (IFN-I) response was observed in both lymphoid and myeloid cells irrespective of COVID-19 severity (**Supplementary Figure S2A**). An evaluation of the transcription factor (TF)-target enrichment for the consensus DEGs consistently revealed significant enrichment of TFs STAT1, STAT2, and IRF9 associated with IFN-I signaling (**Supplementary Figure S2B**). Several RNA-binding proteins (RBPs) were also enriched within the consensus gene set (**Supplementary Figure S2C**, see *Materials and Methods*). Particularly ADAR (IFN-I induced) and YWHAZ were ubiquitously and significantly differentially expressed across severities. ADAR1 is multifunctional and has been extensively studied in the context of innate immunity (22), while YWHAZ expression in SARS-CoV-2 infection has been hypothesized to contribute to the associated neurological deficits seen in COVID-19 (23). Second, lymphoid cells showed an upregulation of cytokines and chemokines/receptors such as CXCR4 and interleukin (IL)-32, in both severe and non-severe samples. Notably, IL-16 was particularly suppressed across multiple lymphoid cell types. Reduced IL-16 levels have been reported in plasma from convalescent COVID-19 patients (24). Third, myeloid cells in both severe and non-severe disease showed an activation of genes influencing myeloid cell differentiation including HIF1A, TRIB1, HCLS1, and chemokine receptors such as CCR1. Several other chemokines and cytokines such as CXCL16, IL1RN, and IL17RA were significantly dysregulated among classical monocytes (CMs) and myeloid DCs (mDCs) but not commonly across all cell types (see *Materials and Methods*). Fourth, within severe disease alone, myeloid cells showed an increased activation of genes associated with leukocyte-mediated immune response and degranulation (25) including annexins and their receptors (ANXA2 and FPR1) and complement receptors such as C5AR1. These cells broadly suppressed expression of genes associated with mitochondrial respiration/oxidative phosphorylation and antigen processing and presentation. Alarmins S100A8 and S100A9, broadly dysregulated across both lineages and severities, showed a consistent upregulation in myeloid cells. Both lymphoid and myeloid cells showed significant dysregulation of immunoglobulin genes including IGHA1/IGHM/IGKC/IGLC2/IGLC3 involved in complement activation, phagocytosis (recognition and engulfment), and regulation of humoral immune response. Interestingly, TRAFD1 and ETV7, negative regulators of IFN-I response, were enriched in severe disease across both cell lineages (**Supplementary Figure S2B**).

In the following sections, we assess several immune subtypes that exhibit significant cell abundance differences between healthy and COVID-19 subjects, including mDCs, MAITs, γδT, PBs, and MKs. We also investigate select subsets within monocytes and NK cells, which contribute to the abovementioned gene signatures and have been less explored (functionally and mechanistically) in COVID-19.

### 2.3 Myeloid Lineage

#### 2.3.1 Monocytes

Monocytes represent a class of hematopoietic-derived innate immune cells whose function can range from inflammatory to anti-inflammatory. Traditionally classified into discrete subsets classical (CD14^high^), non-classical (CD16^high^), or intermediate monocytes (CD14^+^CD16^+^), monocytes are increasingly acknowledged for their cellular, molecular, and functional plasticity (26, 27). Concurrently, current research in COVID-19 has highlighted the overactivation of CD14^+^ monocytes and the emergence of novel monocytic subsets including myeloid-derived suppressor cell (MDSC)-like suppressive monocytes within COVID-19 patients (5, 12, 21, 28, 29). Acknowledging this heterogeneity, we sought to functionally characterize the monocytes focusing on novel subsets and distinguishing them between severe and non-severe COVID-19 (5).

##### 2.3.1.1 Expansion of Both Immunosuppressive and Proinflammatory Subsets

**Figure 2:**
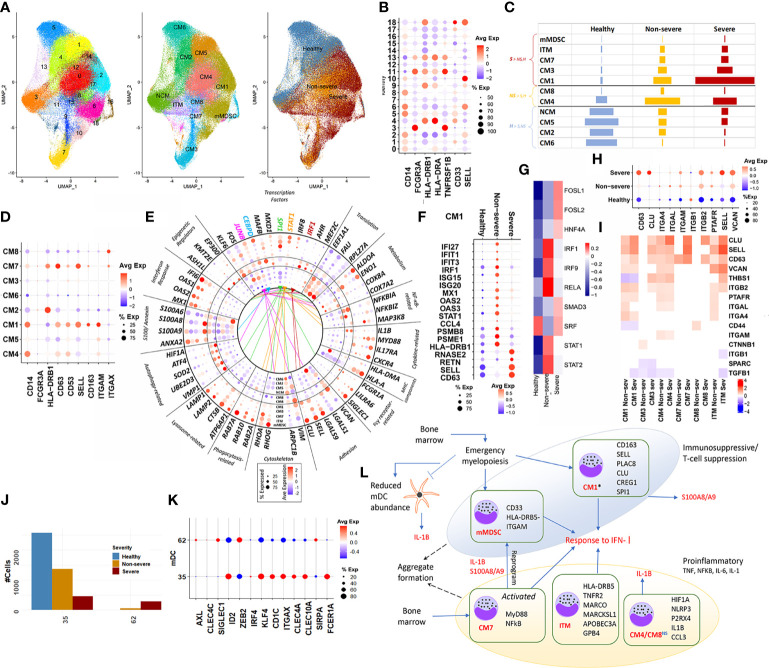
Classical monocytes and myeloid dendritic cells. **(A)** UMAP embedding of monocytes; subset from the original Seurat object. The first of the three embeddings capture the 18 clusters identified; the next captures the grouping of these clusters into distinct subsets, NCM (non-classical monocyte), ITM (intermediate monocyte), CM1–CM8 (classical monocytes), and mMDSC (myeloid-derived suppressor cells). The final embedding captures the severity of the cells. **(B)** Indicates a dotplot of the average expression of the major markers, which are used to classify monocyte subsets into CM, NCM, ITM, and mMDSC. **(C)** Relative cell abundances for each of the 10 subsets identified within healthy, severe, and non-severe subsets. **(D)** Average expression dotplot of major markers identified within classical monocytes that were used to further characterize the CM subsets. **(E)** Average expression dotplot highlighting the expression of several known gene marker involved in various aspects of monocyte functioning. The outermost five concentric rings in the circle plot correspond to the subsets who each have more cells from severe samples (mMDSC, ITM, CM7, CM3, and CM1), middle two rings (CM4 and CM8) have more cells from non-severe samples, and inner four rings (NCM, CM5, CM2, and CM6) have more cells from healthy samples. Colored lines indicate the transcription factor (TF) targets expressed within each subset. **(F)** Dotplot highlights the differences in average expression of cluster markers involved in interferon signaling and degranulation within CM1 alone, across severities. **(G)** DoRothEA TF analysis for cells from CM1 identified differential activity for TFs such as FOSL1, FOSL2, and SMAD3 particularly within severe disease. **(H)** For the subsets with increased abundance in COVID-19 including CM1, CM3, CM4, CM7, CM8, and ITM, expression dotplot highlights increased activity for markers associated with monocytic adhesion migration and signaling. **(I)** For the same subsets as in panel H, the heatmap highlights fold changes for genes that have been previously implicated in the formation of monocyte doublets within pathology. **(J)** Myeloid dendritic cell (mDC) clusters and their count distribution across severity. **(K)** mDCs identified in the integrated dataset represent a mix of conventional DCs (cDC2 and CD1C^+^ DCs) (cluster 35) and pre-DCs (cluster 62). **(L)** A functional map that highlights the role of these various subsets. Relevant cluster markers are highlighted for each subset. We identified two distinct phenotypes associated with the monocytic subsets-immunosuppressive and proinflammatory. The * by CM1, a suppressive subset, indicates the differential interferon type I (IFN-I) responses between severe and non-severe disease, in particular, a more suppressed IFN response within severe subsets due to likely action of repressive factors including FOSL1 (seen in G above). While the theme of interferon response is shared by the subsets, the more nuanced analysis informs us that CM1 and mMDSC lead to immunosuppressive, specifically T cell-suppressive phenotype. CM1 also expressed genes indicative of emergency myelopoiesis in severe coronavirus disease 2019 (COVID-19) infection. Several CM classes share homotypic aggregation and express genes contributing to platelet-monocyte aggregation, thus leading to the hypercoagulability phenotype in severe infection. IL-1 dynamics within each of these subsets were particularly interesting, with increased expression within non-severe disease for CM and mDCs indicative of a dysfunctional mDC state within severe COVID-19.

We subsampled and reclustered 22 monocyte clusters (CD14^+^, CD16^+^, and ITM) from the parent Seurat object, resulting in a total of 19 clusters (**Figures 2A, B**, **Supplementary Figure S3**). Using automatic annotation (SingleR), expression of known monocyte markers, and the cluster markers (**Supplementary Tables S7, S8**), we reannotated the subsampled monocyte space, identifying 11 distinct monocytic subsets including monocytic MDSCs (mMDSCs; **Figure 2C**). Details of transcriptional signatures defining each subset are described in the *Materials and Methods* (Section 4.8). Eight of 11 (CM1–CM8) subsets were identified to be CD14^+^ (**Figure 2D**), of which two subsets were particularly interesting and are discussed below.

Subset CM1 (clusters 2, 6, 10, and 14) was characterized by an increased expression of SELL, alarmins (S100A8/9), CD11b/ITGAM, and CD163^+^. Notably, these clusters do not show a loss of HLA-DR expression but express at levels lower than mean (p.adj < 0.05), as compared with other clusters. This observation is consistent with published research, which has observed a distinct upward shift in the S100^high^ HLA-DR^low^ monocytic population with increasing severity (5, 6, 13). This subset particularly has been suggested to be influenced by and contribute to both the inflammatory proteomic and altered metabolomic plasma profiles seen in severe disease. An evaluation of the cluster markers within CM1 indicated increased expression of S100A8/A9/A12, CD163, PLAC8, ALOX5AP, CLU, SELL, CREG1, and VCAN, as compared with other clusters (**Figure 2E**). These markers have been previously identified to define a unique monocytic subset (MS1) among bacterial sepsis patients and in COVID-19 and may arise from hematopoietic progenitors *via* emergency myelopoiesis, with a potentially suppressive function in pathology (28). TF-target enrichment of all cluster markers within CM1 identified SPI1 (PU.1) as a potent regulator of this subset. CM7 (cluster 9) expressed markers associated with an activated inflammatory state, including CD63, SELL/CD62L, HLA-DR, and ITGAX/CD11c (**Figure 2D**). Notably, this subset expressed MYD88, RELA, and EIF2AK2 (**Figure 2E**). Signaling through the adaptor protein MYD88 (an essential transducer for IL-1B and Toll-like receptor pathways) and the subsequent IFN-I stimulation has been implicated in conditioning of MDSC differentiation during sepsis (30). The frequency of subsets CM1, CM3, and CM7 was increased in severe compared with non-severe COVID-19. Among CM subsets with increased abundances in non-severe disease, CM4 (clusters 0 and 8) and CM8 (cluster 15) exhibited upregulation of cluster markers associated with IL-1β response including HIF1A, NLRP3, EGR1, ICAM1, CCL3, RIPK2, and ANXA2 (p.adj < 0.05). Constitutively expressed HIF1A plays a role in functional re-programming of monocytes from proinflammatory to an immune suppressive phenotype, *via* its regulation of IRAK3 (31).

Differential gene expression analysis (DGEA) of CM1 and expanded subsets in severe disease (CM7, ITMs, and mMDSC) showed significant enrichment of proinflammatory markers and response to IFN-Is (p.adj < 0.05). CM1 and ITM showed a suppression of genes associated with the mitochondrial respirasome including complex I (such as NDUFA11, NDUFB1, NDUFB11, NDUFB7, NDUFC2, and NDUFV2), complex IV (COX4I1, COX7A2, and COX7C), and mitochondrial metabolism including TGFB1, GUK1, PGAM1, UQCR11, and UQCRB, suggestive of considerable metabolic remodeling associated with these subsets in both severe and non-severe disease (**Supplementary Figures S3C, D**).

##### 2.3.1.2 Expanded CM1 Subset Has an Attenuated Response to Interferon Type I in Severe Disease Compared With Non-Severe Disease

CM1 represented the largest subset of monocytes within severe COVID-19 samples, accounting for 46% of all cells from severe disease (**Supplementary Table S8**). Given the marked expansion of CM1 in severe compared with non-severe disease, we sought to delineate the transcriptional differences by performing DGEA between severe and non-severe cells in CM1 alone. Interestingly, CM1 elicited a more potent suppression of genes responding to IFN-I/II (IFI27, IFIT1, IFIT3, IRF1, ISG15, ISG20, MX1, OAS2, OAS3, STAT1, CCL4, CCL5, and FCGR1A) but upregulated gene targets associated with neutrophil degranulation (RNASE2, ANXA2, CD63, KLF4, KLF2, RETN, C5AR1, SELL, and CLU) in severe compared with non-severe disease (**Figure 2F**, **Supplementary Figure S3E**) Additionally, several members of the proteasomal degradation pathway including PSMB8/9/10, PSME1/2, UBC, RNF213, and UBE2LG were suppressed within severe disease for this subset. TF-target analysis revealed increased STAT/IRF activity in non-severe patients and an increased activity for FOSL1, FOSL2, and SMAD3 in severe patients (**Figure 2G**). Increased viremia has been previously reported in FOSL1 knockout chimeric mouse. In the presence of increased viremia and IFN-I production, FOSL1 has been shown to serve as negative feedback inhibiting expression of IFN-I (32). On the other hand, FOSL2 and SMAD3 have been previously shown to cooperatively regulate TGFβ signaling (33). TGFβ can also suppress IFN-I responses by disrupting mitochondrial bioenergetics in alveolar macrophages during respiratory viral infections (34). Taken together, these evidences emphasize the suppression of IFN-I responses in CM1 monocytes especially within severe subjects compared with non-severe subjects.

##### 2.3.1.3 Monocytes Exhibit an Aggregation Phenotype in COVID-19

We observed significant enrichment of gene programs associated with homotypic cell adhesion among several subsets of CMs. CD63 (tetraspanin), which is associated with platelet/neutrophil degranulation and intracellular protein trafficking within monocytes, and L-selectin or SELL (CD62L), which is a major regulator of monocytic adhesion migration and signaling, were significantly upregulated in COVID-19, irrespective of severity (**Figure 2H**). Patients with certain autoimmune conditions (35) and viral infections (36) are known to exhibit increased circulating monocyte aggregates (in both the presence and absence of platelets), which are characterized by an overexpression of SELL and CD63, among other markers. We identified several of the other previously documented monocyte aggregate markers within our analysis including VCAN, and integrins such as ITGA4, ITGAM, and ITGB2 (**Figure 2I**). Additionally, the interaction between platelet specific isoforms of selectin (P-selectin) and CD63 has been recently implicated in the increased formation of platelet-monocyte aggregates contributing to the hypercoagulability phenotype seen in severe COVID-19 (37).

#### 2.3.2 Myeloid Dendritic Cells

Two clusters within the primary Seurat object were identified as mDCs. Using previously published markers of human DCs (38), we identified the mDC subset to represent a mix of conventional DCs (cDC2, CD1C^+^ DCs) (cluster 35 with transcriptional activation of ID2, ZEB2, IRF4, KLF4, CD1C, ITGAX, CLEC4A, CLEC10A, SIRPA, and FCER1A) and pre-DCs (cluster 62, AXL/CLEC4C(CD303)/SIGLEC1), which are typically poised towards cDC2 (**Figures 2J, K**). Relative cDC2 cell abundances reduced with increasing severity (2,577 cells from healthy subjects, 1,426 cells from non-severe subjects, and 734 from severe subjects), consistent with recent observations in severe COVID-19 (39) (**Figure 1D**). DGEA with respect to healthy subjects indicated a robust activation of interferon-stimulated gene (ISG) signatures and response to IFN-I, as observed in monocytes, irrespective of severity (**Supplementary Figure S3F**). Notably, we observed a suppression of key cDC2 mDC markers involved in DC signaling and response within severe disease compared with non-severe disease (**Supplementary Table S9**) including CD83 [marker for activation of mature mDCs (40)], NR4A2 [expressed in immunogenic DCs and promotes anti-inflammatory cytokines (41)], and FCER1A [inflammatory mediator likely promotes immune homeostasis (42)].The mDC also exhibited increased expression of IL-1B in non-severe compared with severe disease (p.adj < 0.05, **Supplementary Table S9**). This suppression of IL-1B expression in mDCs has been suggested to lead to increased production by peripheral monocytes (43) (**Figure 2E**). IL-1β production in both monocytes and mDCs involves the canonical NLRP3 inflammasome-induced IL-1β cleavage and release, dependent on purinergic receptors such as P2RX4 (44). These subsets exclusively expressed purinergic receptors such as P2RX4 (p.adj > 0.05). The impaired IL-1β dynamics arising in severe compared with non-severe disease in addition to the suppression of key markers involved in DC signaling are indicative of a dysfunctional role for mDCs within severe patients. An integrated perspective on the CM and mDC subsets and their phenotypic contributions is provided in **Figure 2L**.

#### 2.3.3 Low-Density Neutrophils

LDN is an umbrella term often used to represent a heterogeneous group of highly adaptable, dynamic neutrophil-like cells composed of a mixture of immature and low-density mature neutrophils, progenitor cells, and granulocytic/polymorphonuclear MDSCs (PMN-MDSC). Given this cellular heterogeneity, their function is suggested to exist on a spectrum, ranging from immunosuppressive to proinflammatory (45, 46).

**Figure 3:**
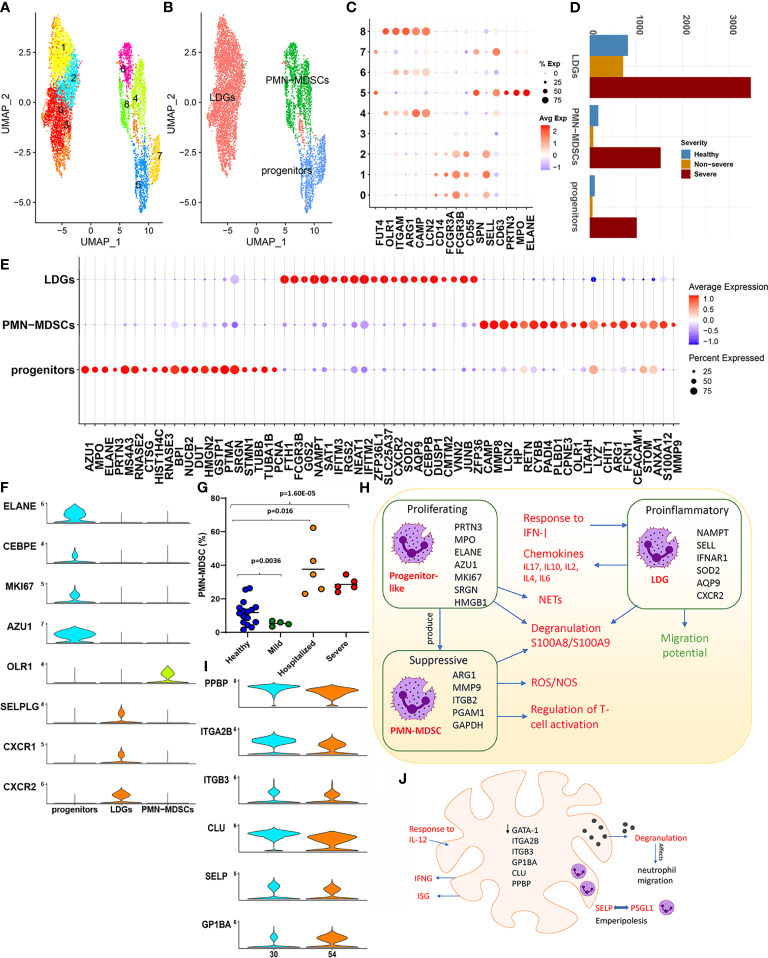
Low-density neutrophils. **(A)** The UMAP embedding of the low-density neutrophils; subset from the original Seurat object after reclustering, highlighting the nine clusters identified. **(B)** This UMAP highlights the grouping of these nine clusters into three distinct neutrophil subsets. **(C)** The dotplot highlights major neutrophil markers identified in the nine clusters, which form the basis for clustering into three distinct groups, namely, low-density granulocytes (LDGs), progenitor-like cells, and polymorphonuclear myeloid-derived suppressor cells (PMN-MDSCs). **(D)** Distribution of the cells from each of the subsets identified. **(E)** The top 20 cluster markers identified within the three distinct cellular subsets. **(F)** Violin plots capture the expression levels of specific genes discussed within the main text, which are specifically expressed in one of the three subsets. **(G)** Cell abundances validated within an independent patient cohort highlight the significant expansion of PMN-MDSCs. **(H)** A functional map of interaction between the three subsets identified within the low-density neutrophils (LDNs). The cluster markers for each subset are highlighted within. Red text indicates increase within COVID-19, and green indicates a reduction. **(I)** Violin plots of markers that define a megakaryocyte population. **(J)** The major functional aspects observed within megakaryocytes (MKs) characterized by GATA-1 low expression and a likelihood for emperipolesis are captured within this representation.

Subsampling and reclustering “LDNs” from the parent Seurat object resulted in nine clusters, grouped subsets into three distinct subsets based on the expression of a combination of established markers, namely, low-density granulocytes (LDGs), PMN-MDSCs, and progenitor-like (**Figures 3A–D**). Details of the transcriptional characterization of these subsets have been provided in Section 4.8).

##### 2.3.3.1 Progenitor-Like Low-Density Neutrophils Are Proliferative and Increased in Severe COVID-19

The progenitor (like) subset exhibited signatures reminiscent of a “proneutrophil” state (5) (**Figure 3E**). We identified this subset to also express AZU1, RNASE3, CSTA, CTSG, and RETN (p.adj < 0.05, **Figure 3E**), which are crucial mediators of neutrophil communication *via* the cytoneme and contribute extensively to NETosis. Interestingly, previous studies have identified several additional markers expressed in this subset including MS4A3, PCNA, HMGN2, SRGN, PTMA, STMN1, DUT, and TUBA1B to be indicative of precursor neutrophils, which differentiate into neutrophils *via* alternate maturation mechanisms (47, 48). This subset also uniquely expressed MKI67 (p.adj < 0.05) suggestive of active proliferation (**Figure 3F**). Taken together, these findings highlight a population of progenitor-like LDNs, which are highly proliferative and activated predominantly in severe COVID-19. Characterizing the transcriptional control (**Supplementary Figure S4A**) revealed increased activity of TFs such as LEF1, MYC, and MYCN within this subset. These TFs are crucial determinants for neutrophil granulopoiesis, proliferation, and differentiation (49), further emphasizing the progenitor-like state for cells contained within this subset.

##### 2.3.3.2 Low-density Granulocytes Respond Potently to Type 1 Interferons, Albeit Muted in Severe Disease and Show Reduced Migration Potential

We observed LDGs to be significantly proinflammatory (expressing chemokines) and IFN-I responsive (**Supplementary Figures S4A–D**) (50). Transcriptional control of LDGs was likewise heavily influenced by TFs such as STAT1/3 and NFKB1/RELA. The top ranked cluster markers identified within LDGs have been previously implicated to play significant roles in neutrophil maturation, activation, and degranulation including NAMPT, IFITM2, IFNAR1, SOD2, G0S2, AQP9, and CXCR2 (**Figure 3E** and **Supplementary Table S10**). Mature neutrophils in certain conditions, such as inflammation and cancer, can present at lower densities, at steady state without additional activation. Notably, activation of AQP9 is associated with changing densities of mature neutrophils due to water uptake, irrespective of activation/degranulation status and their subsequent shift to lower densities (45, 51).

A robust IFN response has been previously reported to originate from LDGs *in vivo* and *in vitro*, with distinct roles in the pathogenesis of diseases such as lupus and malaria (52, 53). DGEA (with reference to healthy cells) indicated that LDGs exclusively had a statistically significant upregulation of genes associated with a potent IFN-I response and activation of ISGs in both severe and non-severe subjects (**Supplementary Table S11**). Dysregulated genes and TFs included BST2, IFI35, IFI6, IFIT1, IFIT2, IFIT3, IFITM1, IFITM2, IFITM3, ISG15, ISG20, MX1, OAS1, OAS3, OASL, RSAD2, XAF1, IRF1, and IRF7. However, this response to interferon signaling seemed to be muted in samples from severe patients compared with non-severe samples. In particular, genes associated with processes involved in FCγ receptor signaling and IFN-I response including CYFIP2, HSP90AA1 HSP90AB1, WIPF1, WAS, ELMO1, CD3G, FYN, PIK3R1, CD247, VAV1, IFI6, OAS2, MX1, and IFIT1 were downregulated in severe disease (p.adj < 0.05). Interestingly, certain proinflammatory ISGs including IFITM1/2/3 and ISG20 were upregulated in severe disease. Alarmins (S100A8/S100A9/S100A12) were upregulated in COVID-19, but significantly higher within severe disease (p.adj < 0.05). Additionally, LDGs within severe samples (compared with both healthy and non-severe subjects) displayed a transcriptional repression of genes associated with neutrophil migration such as CXCR2, CXCR1 (**Supplementary Figure S4E**), CD74, ITGB2, and RAC2 (p.adj < 0.05, **Supplementary Table S12**). CXCR2/CXCR1 serve as cognate neutrophil receptors driving neutrophil migration and are markers of mature neutrophils. In severe sepsis, nitric oxide-mediated suppression of CXCR2 is associated with an impaired migration of neutrophils to the infection loci (54).

##### 2.3.3.3 Polymorphonuclear Myeloid-Derived Suppressor Cells Predominate the Myeloid-Derived Suppressor Cell Response and Contribute to Increased Oxidative Stress Within Severe COVID-19

PMN-MDSCs comprise a functionally distinct phenotype of MDSCs with a neutrophil-like morphology that are known to play important roles in the immune dysregulation of several inflammatory states such as sepsis, cancer, and, as shown here, in severe COVID-19. We confirmed drastic expansion of PMN-MDSCs in an independent cohort of severe and hospitalized COVID-19 patients (**Figures 3D, G**). Given this expansion, we compared cells from severe and healthy subjects to establish the transcriptional landscape. Enrichment of DEGs (**Supplementary Figure S4F**) revealed an expected suppression of genes, which regulate T-cell activation including CD47 (a neutrophil membrane protein), ARG1, CEACAM1, LILRB2, FYN, LYN, ITK, HLA-DPA1/B1, RAC2, PYCARD, RUNX3, ANXA1, ITGB2, and CCL5. The T cell- suppressive activity of PMN-MDSCs is suggested to be driven by a potent induction of reactive oxygen species (ROS), by the nicotinamide adenine dinucleotide phosphate (NADPH) oxidase system and neutrophil degranulation (55). We consistently observed an increased expression of the NADPH generating superoxide genes such as NCF1 and CYBA among highly ranked DEGs (**Supplementary Table S13**) and increased regulation of electron transport chain genes COX5B, NDUFB7, UQCR11, COX6A1, and COX4I1, which participate in oxidative phosphorylation (OXPHOS). PMN-MDSCs in severe samples showed an upregulation of PGAM1 and GAPDH, suggesting increased oxygen consumption due to increased mitochondrial/metabolic energy metabolism *via* OXPHOS, and increased production of ROS (hydrogen peroxide (H_2_O_2_), superoxide anions, and hydroxyl radicals through the NADPH system). Enrichment analysis also indicated a significant enrichment of *S*-nitrosylation within PMN-MDSCs (**Supplementary Figure S4F**). *S*-nitrosylation has been suggested to have inflammatory consequences through a complex interplay of mechanisms especially within pathologies such as sepsis and cancer (56). Interestingly, function of GAPDH, a well-studied target of *S*-nitrosylation, is mediated by alarmins S100A8/S100A9. **Figure 3H** captures the interplay of mechanisms highlighted in the sections above for proliferating progenitor-like, suppressive PMN-MDSCs and the proinflammatory LDGs, particularly within severe COVID-19.

#### 2.3.4 Megakaryocyte Expansion and Evidence for Emperipolesis Within Severe COVID-19 

MKs are the mature cells from which platelets are derived. Recent evidence suggests a direct role for MKs in viral infections (in part *via* IFITM3 upregulation) and in systemic inflammation, highlighting the importance of MKs and their interactions with other immune cells (57). Two clusters from the parent Seurat object, 30 and 54, were annotated as MKs (see *Materials and Methods*) using previously published MK markers (**Figure 3I**). Extravasation to and expansion of MKs among PBMCs (6) and lungs (58) in severe COVID-19 has been recently reported. We concurrently observed a dramatic expansion of MKs within severe samples in the integrated dataset (1,195 healthy cells, 1,153 non-severe cells, and 4,848 severe cells). DGEA in severe and non-severe disease compared with healthy subjects identified 434 and 304 DEGs in severe and non-severe disease, respectively (**Supplementary Table S14**). Notably, both severe and non-severe samples upregulated ISGs including ISG15, IFITM3, and IFI6 and certain immunoglobulins including IGKC, IGLC2, and IGHA1. In severe disease, however, uniquely upregulated genes associated with increased mitochondrial energetics (genes such as COX4I1/5B/6A1/7A2/8A, ISCU, NDUFA1/A2/B1/B11/B3/UQCR11, SOD1/2, and CFL1), leukocyte degranulation (such as S100A8/A9, cathepsins including CTSD/W and SELP), and coagulation (such as FLNA, ITGA2B, ANXA5, MMRN1, and ANO6), alluding to their role in contributing to the thromboembolic phenotype of severe COVID-19 (**Supplementary Figure S4G, Supplementary Table S15**). Severe samples were also enriched for genes associated with an increased response to IL-12, a proinflammatory cytokine. JAK-STAT activation and subsequent IFN-γ generation, after IL-12 stimulation, have been previously reported in patients with immune thrombocytopenia (59).

Thrombocytopenia has been reported as a prominent feature of severe COVID-19 (60). Immune thrombocytopenia is associated with increased platelet demand and additionally exhibit emperipolesis (61). We hypothesized that thrombocytopenia seen in COVID-19 could be associated with the observed MK expansion and MK-mediated emperipolesis. Emperipolesis is a unique phenomenon of cell–cell interaction, which involves a bidirectional membrane transfer between cells such as neutrophils and MKs, where neutrophils enter the MK, fuse their membranes with the MK’s demarcation membrane system (DMS), and then exit the MKs intact. Two receptor ligand pairs have been suggested to mediate emperipolesis including ICAM1/EZR and CD62P/PSGL1 (61). DGEA identified significant upregulation of SELP(CD62P) in severe (compared to healthy) (**Supplementary Figure S4G**). In pathological conditions such as idiopathic myelofibrosis, characterized by emperipolesis, CD62P (a granule protein) is increased and distributed abnormally to the DMS. The abnormal distribution of CD62P to the DMS is suggested to likely trap neutrophils on the DMS *via* its binding to PSGL-1 expressed on neutrophils. Notably, PSGL-1 expression was significantly upregulated in neutrophil subsets, especially LDGs, in severe COVID-19 samples within our study (**Figure 3F**). Emperipolesis is also a prominent feature of MKs in GATA1^low^ murine models, which also exhibit severe thrombocytopenia (62). Consistent with these observations, samples from severe disease exhibited lower (than mean) levels of GATA1 expression (**Supplementary Figure S4H**). Though much remains to be understood on whether emperipolesis is the cause or consequence of thrombocytopenia, current evidence suggests a potential role in the pathogenesis of COVID-19 (**Figure 3J**).

### 2.4 Lymphoid Lineage

#### 2.4.1 Natural Killer Cells

NK cells are a type of lymphocytes that respond rapidly to eliminate/control a host of pathological insults including tumor progression, microbes, and viruses. Recent research in COVID-19 has indicated a robust activation of NK cells, albeit with impaired cytolytic potential and reduced absolute cell counts, with increasing severity (9, 63). Moreover, NK cells also show signs of exhaustion and increased interferon signaling with increased expression of the inhibitory surface proteins in moderate and severe patients (13). The changing NK landscape in COVID-19 has been largely discussed in the context of CD56^dim^/CD56^bright^ (NCAM1) NK cells. However, NK cell populations are increasingly acknowledged to be transcriptionally heterogeneous (64, 65). We subset the NK cells to better characterize their heterogeneity and role within severe and non-severe COVID-19.

##### 2.4.1.1 Characterizing the Heterogeneity of the Natural Killer Cell Populations Identifies Novel NK Subsets

**Figure 4:**
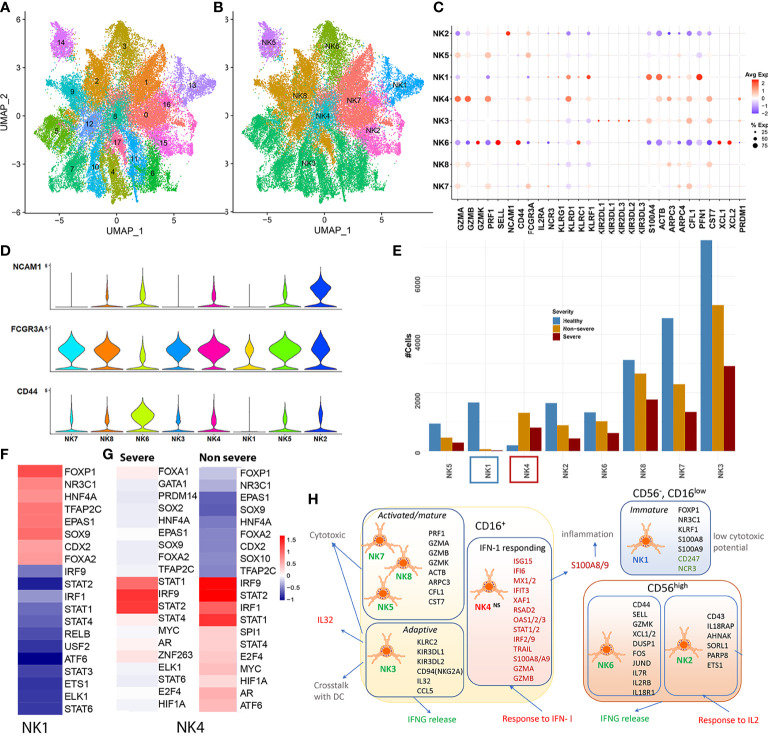
Natural killer cells. **(A)** UMAP embedding of the natural killer (NK) cells after reclustering the subset from the original Seurat object. The first of the two embeddings capture the 18 clusters identified. **(B)** The grouping of the 18 clusters into distinct subsets, NK1–NK7. **(C)** The dotplot of average expression of the major markers that were used to classify NK cell subsets including genes such as the lytic granules, such as granzymes (GZMB/K/A), PRF1; inhibitory KIRs; negative regulators including PFN1 and CST7; transcription factors (TFs) such as PRDM1 and cytoskeletal proteins including ACTB, ARPC3/4. **(D)** The expression of three major surface markers that define NK cell maturity including CD56 (NCAM1), CD16 (FCGR3A), and CD44. NK3–5 and NK7–8 represent a CD56^low^ CD16^+^; NK2 and NK6 were identified as CD56^+^. NK1 was identified to represent a unique group of cells that lacked CD56 expression and had low CD16 expression. **(E)** Cell count distribution of the NK subsets NK1–7. NK1 (blue box) is the only subset with severe abundance loss within disease, while NK4 (red box) is the only subset that has increased abundances within disease. **(F)** DoRothEA TF-target enrichment performed on all cells from the NK1 cluster highlights an increased activity of early TFs such as SOX9, FOXP1, FOXA2, and NR3C1, suggestive of a more precursor/immature like cell state. **(G)** DoRothEA analysis on severe and non-severe cells in subset NK4 highlights the increased activity of STAT1/2 and IRF1/9 in keeping with the increased interferon response seen from NK4. **(H)** A functional map of the NK subsets identified within coronavirus disease 2019 (COVID-19), with cluster markers represented within each subset. NK1 (CD56^−^CD16^low^) represented a unique subset of cells, mostly seen in healthy and lost within COVID-19. These cells exhibit reduced NCR expression, subsequently implying a reduced cytotoxic potential and ability to communicate T and neutrophils. Within the CD16^+^ subsets, we identified an expanded subset that responded potently to interferon type I (IFN-I). The cluster markers of this subset were also significantly differentially expressed in severe and non-severe compared with healthy subjects. Red text indicates increased activity, while green text indicates reduced activity.

The primary UMAP of the integrated dataset indicated a reduction in the NK cell population in severe compared with both non-severe COVID-19 and healthy subjects (**Supplementary Figure S1D**). Reclustering and re-embedding the subsampled NK cells after processing resulted in 18 clusters grouped into seven distinct subsets (**Figures 4A–C**). Subset annotation has been described in detail within Section 4.8 (**Supplementary Figures S5A, B**). Majority of the subsets identified here, including NK3, NK4, NK5, NK7, and NK8, were CD56^low^ CD16^+^ and accounted for nearly 82% of the NK cells identified (**Figure 4D**). CD56^low^ NK cells represent the major circulatory subset of human PBMCs (**Figure 4E**). On the other hand, two subsets NK6 and NK2 were characterized by increased expression of CD56. Both the CD56^+^ subsets expressed PRF1, PRDM1, and S100A4 (a maturation marker) lower than mean (**Figure 4C**). CD56^+^ cells are less cytotoxic but actively produce cytokines such as IFNγ in response to cytokine-mediated stimulation with IL-2 and IL-18. Reduction of CD56^+^ cells, similar to what is observed here (**Figure 4E**), has been implicated in impaired IFN-γ production and reduced cytotoxic activity in certain disease states (66). Particularly interesting however was that the transcriptional profiles of NK2 differed significantly from those of the typical CD56^bright^ (NK6) population. This raised the question if this subset represented an intermediate stage in the maturation of NK cells or a novel subset of CD56^+^ cells with alternate roles. The top cluster markers for NK2 included genes such as NCAM1(CD56), IL18RAP, SPN (CD43), MACF1, AHNAK BTN3A1, SPOCK2, SORL1, PARP8, and ETS1 and functionally enriched for a potent response to IL2 (p.adj < 0.05).

Subset NK1 (cluster 13) on the other hand exhibited a low expression of CD16, along with a lack of CD56/CD44 expression (**Supplementary Figure S5C**). Based on the increased mean expression of KLRF1 (Nkp80) and lack of CD56 expression, NK1 was determined to be a CD56^neg^ CD16^low^ population of NK cells (67). There was a reduced representation of this subset within severe (21 cells) and non-severe (64 cells) compared with healthy cells (1,665 cells from 39 samples). This was particularly interesting because, in contrast, research on other viral and bacterial infections has indicated an expansion of the “dysfunctional and immature” CD56^neg^/CD16^low^ NK population (68). Cluster markers for this set included significant upregulation of alarmins (S100A8/A9), cytoplasmic ribosomal proteins, and OXPHOS genes (**Supplementary Figure S5E, Supplementary Table S16**). NK1 also showed reduced expression of cytotoxicity receptors of active and resting NK cells such as NCR3 (NKp30, which is also involved in interactions between NK cells and neutrophils) and CD247 (CD3ζ, a key subunit of natural cytotoxicity receptors suggestive of reduced cytotoxic potential) (**Supplementary Figure S5F**). Regulon analysis for NK1 cells indicated increased activity of targets of repressive TFs such as FOXP1 [which inhibits JUN/MYC signaling and GZMB and IFNG production in T cells (69)], NR3C1 (glucocorticoid receptor involved in inhibiting IFNG production), and early NK cell TFs such as SOX9, suggestive of a precursor-like, immature state of cells (**Figure 4F**). Whether this subset represents a precursor-like immature population, which is subsequently lost due to maturation within COVID-19, warrants further research.

##### 2.4.1.2 A NK Cell Subset Responds Potently to Interferons

The top cluster markers and differential expression analysis comparing NK4 cells from severe and non-severe patients to healthy subjects indicated a strong activation of genes associated with IFN-I response including ISG15, IFI6, MX1, IFIT3, XAF1, RSAD2, OAS3, and STAT1 (enrichment shown in **Supplementary Figure S5F** and **Supplementary Table S17**). TF activity analysis indicated a robust regulation by IRF2/IRF9 and STAT1/2 for this cluster of cells, within both severe and non-severe COVID-19 (**Figure 4G**). The NK4 subset also showed higher than mean expression of markers including CX3CR1 and CD38, previously attributed to terminally differentiated NK cells (64). NK4 was the only subset to expand within COVID-19 (more prominently in non-severe) (**Figure 4B**). NK4 cells in COVID-19 exhibited cytolytic potential with increased expression of granzymes (GZMB and GZMA) and expressed CD56 (**Supplementary Figure S5G**). In addition to a potent response to IFN-I, DGEA indicated a potent upregulation of alarmins such as S100A8 and TFs such as PLSCR1 in both severe and non-severe subjects, similar to what is seen within the interferon responding clusters of monocytes and LDNs. Notably, in the context of MCMV viral infection, a novel IFN-I dependent mechanism has been identified by which NK cells evade mechanisms of cell death *via* BCL2 signaling (70). The observed expansion of this NK4 subset, in COVID-19, could occur *via* similar pro-survival mechanisms. We outline the abovementioned observations for all the NK cell subsets in **Figure 4H**.

#### 2.4.2 T Cells

##### 2.4.2.1 Characterizing the CD4/CD8 T-Cell Milieu

**Figure 5:**
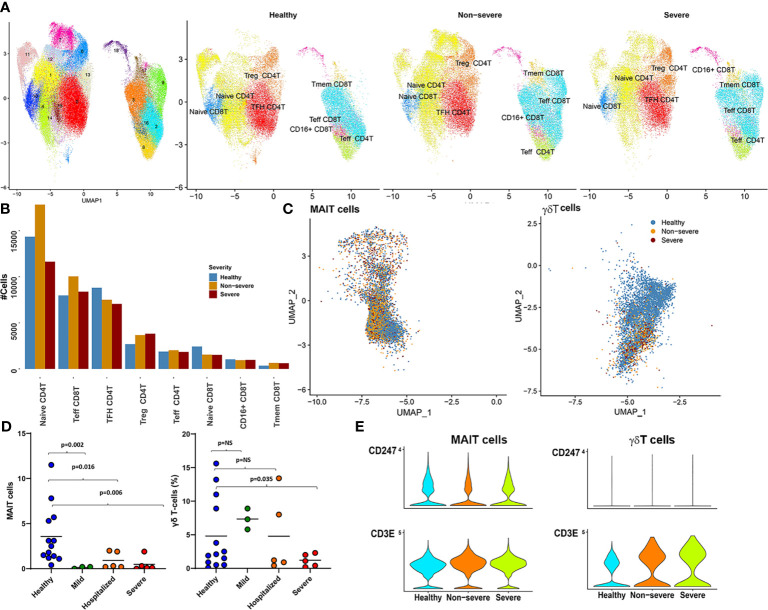
CD4T/8T, mucosal-associated invariant T (MAIT), and gamma-delta T (γδT) cells. **(A)** The UMAP highlights the 18 clusters retained after subsetting CD4T and CD8T cells from parent Seurat object. The right panel shows a side-by-side UMAP of distribution of the various T subtypes (arrived at by grouping cluster based on expression of key factors as identified; see **Supplementary Figure S6B**) in severe, non-severe, and healthy donors. **(B)** A barplot highlighting the abundance differences for each of the eight subtypes identified here across severe, non-severe, and healthy samples. **(C)** The UMAP embeddings of only the MAIT and γδT cells from the original Seurat object. Colors highlight the severities of the cell captured within each cell type. As highlighted also in **Figure 1D**, γδT cells had severely reduced abundances within disease. **(D)** Immunophenotyping revealed similar reductions in MAIT and γδT cell population within an independent patient cohort of coronavirus disease 2019 (COVID-19) and patients exhibiting varying levels of severity. **(E)** Violin plots highlight the increased expression of CD3E and a suppression of CD3ζ (CD247) within COVID-19, for both γδT and MAIT cells, consistent with expression patterns seen within MAIT and γδT in the presence of neutrophils. Suppression of CD247 chain further indicates compromised T-cell signaling without change to T-cell viability.

Adaptive immunity is crucial for successful viral clearance and long-term immune memory, particularly T cells. As noted in the parent Seurat object, CD4T and CD8T subsets did not show very drastic changes between severe, non-severe, and healthy subjects (**Figures 5A, B**, **Supplementary Figures S1D** and **S6C**). To further delineate CD4/CD8T subsets, which might show differential signatures, we subset the CD4T/CD8T populations and reclustered them into eight distinct subsets as described within the *Materials and Methods* (Section 4.8). Abundance signatures of several CD4/CD8T compartments identified here indicated a downward shift, albeit not drastic, with increasing severity (**Figure 5B**), which is consistent with much of the published research (5, 7). Specifically, we observed a higher proportion of CD8T/CD4T effector (Teff) cells in COVID-19 compared with healthy subjects, but lower in severe compared with non-severe subjects. A higher percentage of activated and proliferative CD8T effector population has been documented within less severe infections (12). Likewise, a large proportion (~36.6%) of the cells identified as naïve CD4T cells were significantly increased in non-severe disease, compared with severe disease, consistent with published research (71). In contrast to the abundance trends of CD4T/8T naïve and effector cells, we observed a progressive increase in the abundances of CD4^+^ Tregs, a low-frequency cell type, across severity (**Figure 5B**). Increase in the CD4^+^ Tregs has been previously reported through flow cytometry experiments in the blood of COVID-19 patients (71). Tregs are crucial for regulating immune homeostasis and autoimmunity, controlling the quality and magnitude of immune responses in infections by modulating expression of key factors including FOXP3, CTLA4, and IL2RA (**Supplementary Figure S6B**) (72).

Particularly interesting within the clusters identified were two clusters 16 and 18, which, in addition to CD8 Teff-like signatures (including expression of granzymes, IFNG, PRF1, and NKG1), showed expression of CD16. The CD16^+^CD8T subset showed significant increases in proportion of cells within both severe and non-severe COVID-19 compared with healthy cells (**Supplementary Figure S6C**). CD16^+^CD8T cells have been reported previously in certain viral infections such as hepatitis (73) and in blood from smokers (74) and are suggested to emerge as a consequence of differentiation of T cells beyond terminally differentiated effector states, acquiring CD16 and NK-cell like functional properties. To further elucidate the functional relevance of this subset in COVID-19, we examined how the transcriptomes of this subset differed compared with those of healthy subjects. DGEA analysis identified 140 DEGs in severe and 201 DEGs in non-severe disease compared with healthy subjects (**Supplementary Table S18**). MSigDB analysis revealed both sets of DEGs to be significantly enriched for NK/T cell repertoires, consistent with the earlier characterization of NK-T-like cells (**Supplementary Figure S6D**). Functional analyses revealed that both severe and non-severe disease subsets exhibit significant response to IFN-I signaling (75) and an overexpression of alarmins including (S100A8/A9) (**Supplementary Figure S6E**). Notably, however, only within severe disease do we see an upregulation of functions typically associated with platelet degranulation, homotypic adhesion, and coagulation including markers such as PPBP, ITGA2B/ITGB3, PF4, and TREML1. While most of these factors are typically associated with myeloid cells, emerging evidence suggests a role for these markers in modulating CD8T and NK differentiation and function in health and disease and is worth exploring in the context of this subsets expressing CD16 and NK-like CD8^+^ T cells.

##### 2.4.2.2 A Muted Response of T-Cell Subsets Including Mucosal-Associated Invariant T and Gamma-Delta T Cells Exists in COVID-19, Likely Contributed to by Increased Neutrophils and Oxidative Stress

A notable decrease in cellular abundances of low-frequency T subsets including MAIT (cluster 15) and γδT (cluster 29) was seen in COVID-19 samples compared with healthy subjects (**Figures 1B**, **5C**, and **Supplementary Figure S1D**). This change in cellular frequencies was further validated in an independent cohort (**Figure 5D**). Each cluster expressed markers consistent with cell types, MAITs expressed (KLRB1 (CD161), SLC4A10, NCR3, DPP4 (CD26), IL7R, and GZMK) (76); and γδT cells expressed transcriptional markers such as CD8A, CD8B, CD2, CD5, CD7, TRDC, and TRGV9. Interestingly, CD26 (DPP4), a suggested target for the SARS-CoV-2 spike proteins, was also found to be expressed only within subsets of the lymphoid compartment, especially MAIT (77) (**Supplementary Figure S7A**).

MAITs are a class of non-conventional T cells, representing 1%–10% of the circulating T-cell population and preferentially respond to innate inflammatory signals including IL-12, IFN-γ, and IL-18 with viral infections including COVID-19 (76, 78). Likewise, γδT cells, which are a class of restricted T cells, are also activated preferentially by IL-12. Consistently, DGEA for MAIT and γδT cells identified an increased response to IL-12, particularly in severe disease (compared with healthy subjects), with an additional enrichment of genes associated with IL-2 production (more prominent in non-severe disease) (**Supplementary Figures S7B, C**). Despite reduced abundances, MAIT cells in severe and non-severe disease showed increased activation [as detected through expression of CD69, an early activation marker (p.adj < 0.05), consistent with earlier reports (78)] (**Supplementary Figure S7D**).

Under inflammatory conditions, γδT recruits and activates neutrophils through the release of cytokines and chemokines. In the presence of H_2_O_2_, neutrophils can suppress γδT action (65). Neutrophils are also thought to suppress MAIT cells in the presence of H_2_O_2_ (66). Increased expression of CD3E and a suppression of CD3ζ (CD247) was observed on MAIT and γδT cells in COVID-19 samples (**Figure 5E**). These expression patterns have been previously observed in MAIT and γδT in the presence of neutrophils. Suppression of CD3ζ chain further indicated compromised T-cell signaling without change to T-cell viability. These observations suggest that the reduced expansion of these cell types in COVID-19 is likely due to excessive activation of neutrophils/LDNs and increased oxidative stress. Whether loss of circulating MAIT and γδT to the airways and other tissues contributes to the observed reduction and subsequent turnover merits a more thorough investigation.

#### 2.4.3 B Cells

In the context of acute viral infections such as in COVID-19, immature B cells mature to naïve B cells and differentiate to memory cells or antibody-secreting cells (ASCs) upon antigen activation. Extant omics analyses of immune remodeling in COVID-19 have shown overall lymphoid dysfunction, with decreases in multiple cell types, including naïve B cells; however, PBs were a notable exception and showed expansion and heterogeneity with increasing disease severity (6, 10, 21). A flow cytometric study of multiple B-cell subtypes showed overall decreases in most B-cell subtypes, with increasing disease severity, except for ASCs (17). Likewise, other previous flow cytometric analyses report expansion of oligoclonal PBs in severe COVID-19 (11, 79). To better understand how intracellular mechanisms, including transcriptional regulation, may contribute to B-cell dysfunction with increasing disease severity, we subset B cells as identified in the parent Seurat object (**Figure 1**) for further analysis (**Supplementary Figure S8**). Of these, we analyzed in greater detail the populations that had the most marked changes between healthy, non-severe, and severe COVID-19 subjects: naïve B cells, which reduced with increasing severity (**Supplementary Figure S8**), and PBs, which expanded in severe COVID-19 (**Figure 1D**).

##### 2.4.3.1 Naïve B Cells Expand Robustly in Non-Severe Disease

**Figure 6:**
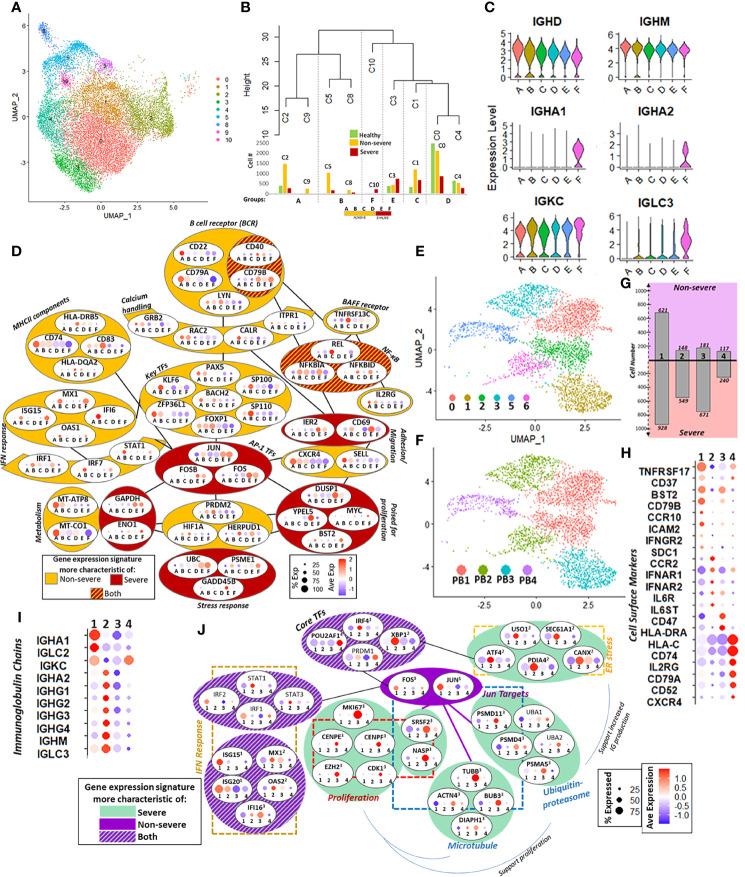
Naïve B cells and plasmablasts (PBs). **(A)** UMAP of subsampled and reclustered naïve B cells (see *Materials and Methods*) with initial clusters 0–6 and 8–10. **(B)** Hierarchical clustering grouped together clusters with similar proportions of healthy, non-severe, and severe cells. The number of cells in each severity category is shown in the corresponding bar graphs under the dendrogram. This resulted in six final groups as summarized in the horizontal box underneath the bar graph: A–F had more healthy and non-severe cells than severe (orange, H, NS > S), and E and F had more severe than healthy, non-severe (red, S > H, NS). **(C)** Violin plot showing expression of selected immunoglobulin heavy and light chains in groups A–F. **(D)** Group-dependent gene expression patterns in several categories that modulate naïve B-cell function and subsequent activation, including B-cell receptor signaling, calcium handling, MHC-II components, B-cell activation factor (BAFF) receptor, interferon (IFN) response, metabolism, stress response, priming for proliferation, adhesion, and transcriptional regulation, including AP-1 transcription factor (TFs). Each oval contains the gene name with percent and average expression of that gene across the six groups A–F. Each gene had significant differential expression (p.adj ≤ 0.05) unless otherwise specified. Genes within dark red colored ovals were expressed more in severe coronavirus disease 2019 (COVID-19); genes within orange ovals were expressed more in non-severe cells; genes in red and orange striped ovals had expression in both severe and non-severe cells. **(E)** Six clusters with surface marker expression most characteristic of PBs (CD19^−^, MS4A1^−^, CD27^+^, and CD38^+^) were retained after subsampling and reclustering the parent Seurat object (see **Supplementary Figures S8F, G**). **(F)** Four PBs subsets (PB1, PB2, PB3, and PB4) were defined from six clusters in **(E)** based on expression of the top cluster markers (**Figure S8H**). **(G)** Bar graphs show number of cells from non-severe (purple) and severe (pink) coronavirus disease 2019 (COVID-19) patients in each PB subset (1 = PB1, 2 = PB2, 3 = PB3, and 4 = PB4). **(H)** Cell surface marker expression across four PB subsets (1 = PB1, 2 = PB2, 3 = PB3, and 4 = PB4) reflects PB population heterogeneity with respect to B-cell receptors, cytokine/chemokine receptors, adhesion molecules, and antigen presentation. **(I)** PB subset-dependent expression of immunoglobulin chain genes (1 = PB1, 2 = PB2, 3 = PB3, and 4 = PB4). **(J)** Subset-dependent expression of core TFs that regulate PB commitment and several downstream targets, including genes involved in mediating endoplasmic reticulum (ER) stress, proteasome function, microtubules, and IFN response. Each oval contains the gene name with percent and average expression of that gene across the four PB subsets (1 = PB1, 2 = PB2, 3 = PB3, and 4 = PB4). Superscript denotes that the gene had significant differential expression (p.adj ≤ 0.05) in that subset relative to the other subsets.

From the initial UMAP (**Figure 6A** and **Supplementary Figure S8**), we identified six groups of naïve B cells (A–F) by unsupervised hierarchical clustering (see *Materials and Methods*) (**Figure 6B**). Groups A–D comprised predominantly of healthy and non-severe cells, while in groups E and F, severe cells outnumbered healthy and non-severe cells. For clarity, groups A–D will hereafter be referred to as “B_NS_” (mostly non-severe), and groups E and F will be “B_S_” (mostly severe). All the groups had expression of IGHD and IGHM, consistent with naïve B cells. Group F has additional expression of IGHA1/2, perhaps indicating priming for early stages of activation (**Figures 6C**). Expression of multiple markers was consistent with naïve B cells, and the top 15 significantly expressed cluster markers for each group of A–F are shown in **Supplementary Figure S8**.

**Figure 6D** summarizes the remodeling of naïve B cells in COVID-19. Signaling *via* the canonical B-cell receptor (BCR) is crucial for B-cell survival, development, and antibody production. Genes involved in BCR signaling had variable expression in non-severe and severe groups; for instance, CD22 and CD79A showed increased expression in B_NS_ groups, while CD40 and CD79B were increased in B_S_. Genes downstream of BCR signaling involved in regulation of intracellular calcium showed increased expression within B_NS_. Several other cell surface markers, including MHC-II components (CD74 and CD83), TNFRSF13C, and IL2RG, showed increased expression in B_NS_ relative to B_S_, while both groups expressed NF-κB inhibitor genes (NFKBIA/D), downstream of TNFRSF13C. Taken together, these indicate a severity-dependent difference in naïve B-cell activation, downstream intracellular signaling, and antigen presentation. Although key TFs for early B-cell maturation (such as PAX5, BACH2, and FOXP1) were expressed in all groups, they were statistically significantly increased expression in B_NS_ groups. In addition to subtle severity-dependent variations in maturity and activation, this could reflect multiple origins for naïve B cells in secondary lymphoid organs. For example, ZPF36L1, a TF required for the maintenance of marginal B cells, is increased B_NS_ (80). By contrast, multiple AP-1 family TFs including JUN, FOS, and FOSB had increased expression in the B_S_. Notably, group B within B_NS_ showed a robust response to interferon stimulation, with increased expression of TFs such as IRF1, IRF7, and STAT1, as well as downstream genes including MX1, OAS1, and IFI6.

We observed a metabolic shift from oxidative phosphorylation to glycolysis with increasing disease severity, which likely reflects the primed state (for activation) of these cells. We additionally observed that B_NS_ had expression of genes protective against oxidative stress compared with B_S_ including HERPUD1, HIF1A, and GADD45B (may be protective against genotoxic stress in lymphocytes) (81), while B_S_ had an increased expression of ubiquitin–proteasome genes (e.g., PSME1 and UBC). Differences in stress response could play a role in preparing B cells in COVID-19 for enhanced proliferation. Pro-proliferation genes such as MYC and BST2 were increased in Bs groups, likely suggestive of a state primed for proliferation in severe disease. Additionally, expression of cells markers related to adhesion and migration including a reduced expression for CXCR4 and SELL in B_S_ with an increased expression of CD69 and IER2 could indicate an increased potential of B cells to differentiate into PBs (82, 83). Taken together, these could indicate a state of naïve B cells primed for differentiation, perhaps more readily to the PB fate, within severe COVID-19.

##### 2.4.3.2 Plasmablast Exhibits Significant Heterogeneity and Expands Drastically Within Severe COVID-19

PBs are short-lived, antibody-producing cells that are derived from antigen-activated memory B cells. We observed considerable expansion and increased heterogeneity of the PB cell population with increasing disease severity (**Figure 1D**). Previous flow cytometric analyses have reported expansion of oligoclonal PBs, notable even in the context of overall lymphoid cell dysfunction, in severe COVID-19 (11, 79). While extant omics analyses of immune dysfunction in COVID-19 have shown PB expansion and heterogeneity (6, 10, 21), the intracellular mechanisms that may contribute to cell dysfunction have not been explored in detail. To this extent, we subsampled and reclustered the PBs from the parent Seurat object resulting in 10 clusters, which were subsequently grouped into four major subsets (described in Section 4.8) (**Figures 6E–G** and **Supplementary Figure S8**).

Subset-dependent expression of cell surface markers, including BCR components, cytokine/chemokine receptors, and HLA genes, reflected putative heterogeneity in cell growth, homing potential, and maturity (**Figure 6H**). Genes involved in promoting PB survival and growth TNFRSF17, BST2, and CD79B had increased expression in PB1, the dominant signature in non-severe patients. Chemokine and cytokine receptors, likewise, had varied expression across subsets including CCR10, ICAM2, and IFN-γ in PB1; CCR2, IFN-α, and IL-6 receptor components in PB2; and CXCR4 and IL2RG in PB4. Increased expression of CCR10 in PB1 (more prevalent in non-severe disease) may be indicative of cells that home to mucosal surfaces (84), while CCR2- and CXCR4-positive cells in PB2/4 (more prevalent in severe disease) may be indicative of cells that home primarily to the bone marrow (85). Immunoglobulin chain expression was also subset-dependent, with IGHA1 more highly expressed in PB1, while PB2 had robust expression of the IGHG chains (**Figure 6I**). Finally, surface marker expression, together with TF expression (**Figure 6J**), suggested varying maturation across PB subsets, with increased expression of IRF4, PRDM1, and XBP1 in PB2/4 indicating increased maturity (86).

We next investigated the subset-dependent expression of key TFs and several of their targets that regulate PB commitment. All subsets expressed the core TFs involved in PB maturation—IRF4, PRDM1, XBP1, and POU2AF1—albeit with differential expression between the subsets: POU2AF1 was significantly expressed in PB4, while IRF4 and XBP1 expression was significantly increased in PB2. Concomitant with XBP1 upregulation in PB2, its downstream targets related to endoplasmic reticulum (ER) stress response, including genes involved in the unfolded protein response such as ATF4, were also significantly upregulated relative to the other subsets (87). TFs JUN and FOS, though expressed in all subsets, were significantly upregulated in PB1. JUN targets involved in proteasome function were expressed in PB1 and significantly expressed in PB3. Together with the ER stress response, protein degradation pathways support increased immunoglobulin production in PBs. The differing significance of expression for JUN/FOS and their targets across PB1 and PB3, respectively, may reflect negative autoregulation of JUN and FOS within each group. We also noted active proliferation, especially in PB3 (more salient in severe samples) with significantly increased expression of MKI67, EZH2, and CDK1. JUN targets involved in cell cycle regulation (such as SRSF2 and NASP) and microtubule-related genes (such as TUBB) were significantly upregulated in PB3 and may also support increased PB proliferation. Finally, we observed a heterogeneous response to IFN-I stimulation across PB subsets. IRF1/2 and STAT1/3 were expressed in groups PB1 and PB2, respectively (albeit p.adj > 0.05). ISGs also had a subset-dependent expression with ISG15/20 significantly upregulated in PB1, and while MX1 and OAS2 were significantly upregulated in PB2. Taken together, our analyses indicated a more heterogeneous PB response, which may reflect more varied maturity and functional status, in severe compared with non-severe COVID-19 patients (**Figure 6J**). Further, while all subsets responded to IFN-I, PBs from severe COVID-19 patients were characterized by robust proliferation and ER stress response, which may support increased immunoglobulin production.

## 3 Discussion

Current research utilizing high-throughput data from either limited cohorts or focused analyses of specific biological systems have generated valuable insights into the pathogenesis of COVID-19. Complementary to these reported observations, in this study, we present system level mechanistic insights into the pathogenesis of COVID-19, by integrating scRNAseq data from four sizable cohorts in both severe and non-severe COVID-19. Integration allowed for higher granularity in identifying and characterizing transcriptomic and cellular heterogeneity of immune response within COVID-19.

**Figure 7:**
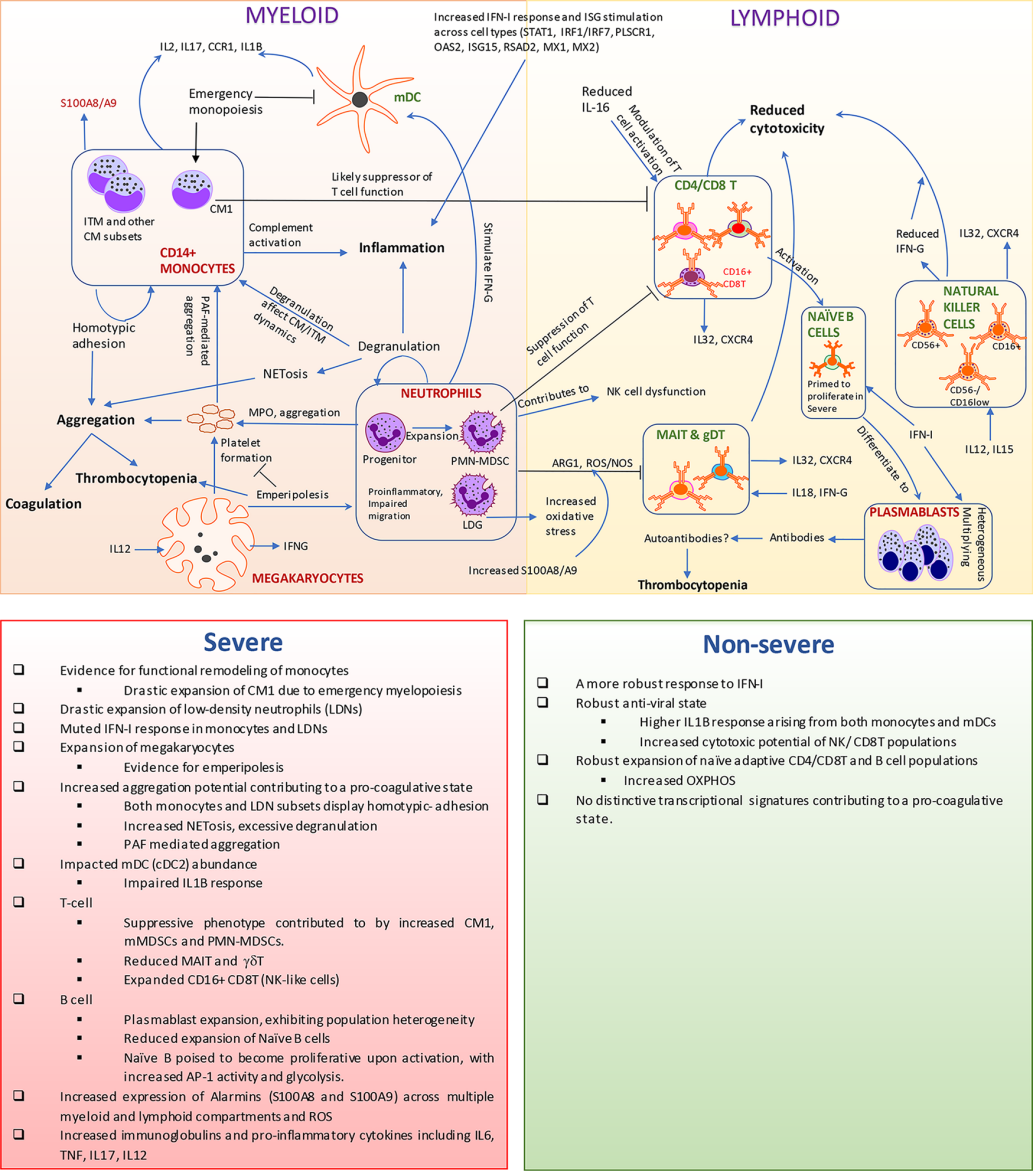
Transcriptomic and cellular heterogeneity of immune response within coronavirus disease 2019 (COVID-19). This figure encapsulates the main results identified within our manuscript. Rapid expansion of specific monocytic subsets, megakaryocyte, plasmablasts, and low-density neutrophils, along with reduction in cellular frequencies of mucosal-associated invariant T (MAIT), gamma-delta T (γδT), and natural killer cells, is seen within COVID-19, particularly severe disease. The observed reduction of myeloid dendritic cells could be a consequence of emergency monopoiesis, which results in the drastic expansion of suppressive subsets such as CM1 monocytes in severe disease. We observe an increased activation of interferon type I (IFN-I) response, interferon-stimulated genes (ISGs), and alarmins arising from both lymphoid and myeloid cells. However, low-density granulocytes (LDGs) and CM1 show a more muted response to IFN-I in severe disease, compared with non-severe disease. The increased oxidative stress along with alarmins likely contributes to the suppression of T cells particularly MAIT and γδT. Neutrophils/LDNs have an increased tendency to spontaneously produce neutrophil extracellular traps (NETs), which has been observed in COVID-19 and suggested to contribute to the coagulopathy in COVID-19. Additionally, both monocytes and LDNs presented transcriptional signatures associated with aggregation, especially in severe COVID-19. The increase in MKs in circulation and likely emperipolesis within COVID-19 adds to the mounting evidence on the potent link between thromboembolic events mediated by platelets and their precursors and neutrophils/LDNs. A potential link between immune thrombocytopenia observed in patients and the expansion of MKs/emperipolesis, increased aggregation potential of various immune cell types, and generation of autoantibodies warrant further investigations especially within severe COVID-19 patients. The accompanying bottom panel highlights all differences observed in our manuscript between severe and non-severe disease.

Modulation of innate immune cells manifested in several ways, distinguishing the responses between severe and non-severe COVID-19 (**Figure 7**). Notably, the systemic and robust upregulation of gene signatures in response to IFN-I occurred across multiple cells within both myeloid and lymphoid lineages in both severe and non-severe disease as detected *via* consensus gene signatures (**Figure 1**) and expression of IFN-I receptors IFNAR1/2 (**Supplementary Figure S9**). Differing reports exist on the activation of a robust IFN-I response in COVID-19 within the PBMCs (88), while there is significant interferon response in lung epithelial cells (2). We observed that specific subsets that expanded significantly in COVID-19 exhibited a more potent response to IFN-I including the NK4 subset within NK cells, CM1 within CMs, LDGs within LDNs, CD16^+^CD8T subset within CD4T/8T, naïve B cells, and PBs. However, this response, specifically in CM1 and LDGs, was more muted in severe compared with non-severe disease. TF factor analysis within these subsets revealed several master regulators, which likely contribute to the observed dysregulation within severe and non-severe disease. Overall, while we cannot comment on the kinetics of IFN-I response, a robust response does exist in COVID-19 albeit muted in severe disease, compared with healthy subjects (12). Increased expression of S100A8/A9 (alarmins) was seen arising from the abovementioned expanded subsets (such as CM1, LDGs, and CD16^+^CD8T) in severe disease. Alarmins have diverse roles and contribute extensively to neutrophil recruitment and degranulation and are increasingly acknowledged as crucial markers for COVID-19 infection (89). The increased oxidative stress arising from LDNs as seen in our analysis [and likely other granulocytes (90)], along with expression of alarmins, could significantly contribute to T-cell suppression including CD4T, CD8T, MAIT, and γδT, especially in severe disease.

Severe inflammation has been suggested to induce a state of emergency myelopoiesis, particularly monopoiesis and granulopoiesis (28). We evidenced a drastic expansion of distinct subsets of MDSC (like) cells arising likely due to emergency myelopoiesis such as CM1 within CMs and PMN-MDSCs (through neutrophil progenitor-like cells) within the LDN subsets, especially in severe patients. In certain bacterial infections, experiments have revealed that infection-induced emergency monopoiesis occurs at the expense of DCs, particularly mDC development, and can be sustained for weeks after pathogen clearance (91). It is therefore possible that the extent of emergency monopoiesis seen in severe COVID-19 (over non-severe COVID-19) contributes to the significant loss of mDCs (p < 0.01, **Supplementary Figure S1D**). This loss of abundance taken together with their transcriptionally dysfunctional state in severe disease warrants further investigations of mDCs in the context of SARS-CoV-2 infections.

Nearly 35% of severe COVID-19 patients experience thromboembolic events at multiple sites including the microvasculature, brain, and lung, which can cause organ failure in these patients. Though the precise mechanisms are unclear, several functional links have been proposed, including an interaction between the plasminogen, complement, and platelet-activating systems in severe disease (2). Both neutrophils and LDNs have an increased tendency to spontaneously produce neutrophil extracellular traps (NETs), which has been observed in COVID-19 and suggested to contribute to coagulopathy in COVID-19 (92, 93). We identified that both monocytes and LDNs presented transcriptional signatures associated with aggregation as well as homotypic adhesion in severe COVID-19. LDGs and CD14^+^ monocytes express PTAFR, which indicates PAF activation (a phospholipid crucial for driving platelet aggregation). LDGs also express SELPLG (PSGL-1), a glycoprotein counter-receptor for the cell adhesion molecules P-, E-, and L-selectin, which are required for tethering of leukocytes to activated platelets or endothelia expressing selectins. MKs were found to express the ligand SELP, suggesting interactions between MKs and LDNs, and an active role for MKs in the pathogenesis of severe COVID-19. Our earlier observations on the likelihood of emperipolesis within COVID-19 adds to the mounting evidence for the potent link between thromboembolic events mediated by platelets (aggregation) and neutrophils/LDNs (2, 92). Current research provides evidence for the role of MK cytoplasm and α-granules as soluble mediators with potential to impact neutrophil migration, behavior, and survival *in vivo* and *in vitro* (61). It is hard to say whether the expansion of MK in severe disease is a cause or effect of increased neutrophil/LDN mobilization and the observed thrombocytopenia. Circulating LDN levels have been reported to correlate with disease severity, contributing to enhanced cytokine production and systemic increases d-dimer, IL-6, and TNF-α levels, in humans with COVID-19 (93, 94). Ratio of circulating γδT to LDN cells has been suggested to correlate with COVID-19 severity and serves as an early marker of severe disease (94). Neutrophils are known to mediate inhibition of NK development, function, and homeostasis (95) while also being influenced by MAIT population levels, for a controlled immune response (76, 96), suggestive of similar roles for LDNs. Unlike other viral infections, we observed no significant changes to majority of the CD4T/CD8T milieu; however, there is evidence for functional exhaustion. Even though there is no significant remodeling of active B cells, naïve B cells are poised for differentiation, and PBs downstream of the B cell maturation spectrum expand drastically within severe COVID-19, in contrast to other respiratory infections.

In summary, our analyses provided novel functional insights into both systemic and cell type-specific changes that contribute significantly to the pathogenicity of COVID-19 in severe and non-severe patients, as shown in **Figure 7**. Our analysis of the differential interferon response within immune cell subsets in severe and non-severe COVID-19 allowed for mechanistic insights that help explain prior work. We further highlighted that LDN responses could play a crucial role in COVID-19 pathogenesis and in distinguishing severe from non-severe responses. Particularly, they can serve as a crucial nexus between various myeloid and lymphoid cells affecting their interaction dynamics and contributing extensively to a proinflammatory and pro-thrombotic phenotype seen in severe COVID-19.

### 3.1 Study Limitations

We acknowledge several limitations of the current study including limited control over patient cohort selection and initial sample processing techniques due to the use of published data. Factors such as definition of disease severity, sampling time after symptom onset, variability in patient treatment conditions, comorbidities, and sex could all affect clinical presentation and subsequent transcriptomic landscape. As our proposed mechanistic framework is based on integrated analysis of scRNAseq-derived transcriptomic signatures from the PBMCs and assessed based on the current understanding of immune cells, future experiments in an independent cohort are required for targeted insights and further validation.

## 4 Materials and Methods

In our current study, we integrated publicly available scRNAseq data derived from PBMCs of COVID-19 (and healthy) patients. The four original publications included Wilk et al. (10), Lee et al. (4), Arunachalam et al. (20), and Schulte-Schrepping et al. (5) (**Supplementary Table S1**). We utilized Seurat for all scRNAseq processes indicated in this study.

### 4.1 Single-Cell RNA Sequencing Data Acquisition and Integration

For data from the PA and Lee studies, where the CellRanger outputs were available, count matrices were constructed using Read10X function, and a Seurat object was subsequently generated using Seurat v3.2 (97). We excluded flu samples from the Lee study prior to Seurat object generation. Likewise, a Seurat object was generated from the Wilk study as indicated in the original publication and its Supplementary Material. However, at the time of data download for our current analysis (September 20, 2020), raw count files were as yet unavailable for download from Schulte-Schrepping et al. (EGAS00001004571). Subsequently, we downloaded processed Seurat objects from fastgenomics.org (files seurat_COVID19_PBMC_cohort1_10x_jonas_FG_2020-08-15.rds and seurat_COVID19_PBMC_jonas_FG_2020-07-23.rds), as indicated in the original manuscript. We extracted the “counts” slot within the Seurat objects and utilized them for downstream integration. Given that the metadata for each study captured varying amounts of information (see **Supplementary Table S2**), we restructured the metadata columns to facilitate downstream integration; specifically, we generated four new columns, “orig.ident”, which captures the donor information from each study. Multiple samples from the same donor were aggregated for the purposes of this analysis: “Infection_status” (levels: Covid, Healthy) captures the condition of cells/samples, “Severity” (levels: Severe, Non-severe and Healthy) captures the severity of diseases among COVID-19 patients, and “Study” captures the dataset name (Wilk, Lee, SS_C1, SS_C2, and PA). All moderate, mild, and asymptomatic (from Lee et al, see **Supplementary Table S2**) donors across studies were reannotated to “Non-severe,” while all “ventilated” donors were annotated to “Severe” (from the Wilk study, **Supplementary Table S2**), for the sake of consistency. Filtering of the data was performed at cell and gene levels, to obtain reduced datasets, which were then used for downstream integration (see *Data Filtering*). Only the 14,063 common features across all five datasets were considered for all downstream analyses. Notably, some genes of interest such as IFNB1 are absent from 14,063 features and subsequently absent within our analysis here.

### 4.2 Data Filtering

For each of the individual datasets identified above, we performed cell-level filtering by excluding cells based on the following quality criteria: more than 25% mitochondrial reads, more than 25% hemoglobin genes (heme reads), less than 250 expressed genes or more 2 std. deviations from the mean, less than 500 detected transcripts, and a complexity (log10 genes/UMI) of less than 80%. Additionally, we performed gene-level filtering by excluding genes that were expressed in less than 10 cells. To facilitate downstream integration, we only utilized the 14,063 features/genes common across all datasets for further analysis. Each sample cell contribution after filtering is captured within S2-meta (**Supplementary Material**).

### 4.3 Data Integration

Each filtered dataset was log normalized prior to integration. The original gene counts for each cell were normalized by total UMI counts, multiplied by 10,000 (TP10K), and then log transformed by log10(TP10k + 1). A total of 2,000 most variable features were detected within each dataset using the vst method implemented in Seurat. Subsequently, the five normalized datasets were integrated using the IntegrateData option of Seurat v3.2, on 2000 gene anchors identified *via* FindIntegrationAnchors (ndims = 30). The final integrated dataset considered for this analysis contained 14,063 features across a total of 375,438 cells from 111 donors (33 severe, 28 non-severe, and 50 healthy subjects, contributing a total of 116,234, 118,023, and 141,181 cells respectively) (**Figure 1A**, **Supplementary Table S3**).

### 4.4 Data Scaling and Dimensionality Reduction

Data scaling and dimensionality reduction (nPCs = 30) were performed on the integrated dataset. Linear regression was performed to remove unwanted variation due to % mitochondrial or heme reads. Optimal dimensions for clustering was identified using “ElbowPlot” function as 24. Subsequently, for two-dimensional data visualization, we performed UMAP based on the first 24 dimensions of the integrated dataset, with the cells clustered using the Louvain algorithm. Given the very large number of cells (~300K), we set the resolution at 2.5 to get meaningful clusters by using the “FindClusters” function.

### 4.5 Establishing the Cellular Cluster Identity of the Integrated Dataset

Cluster identity of the integrated Seurat object was established and confirmed using a threefold strategy. We first ran SingleR, an automatic cluster annotation tool that compares the transcriptome of each single cell to reference datasets to determine cellular identity. We utilized a combination of SingleR’s inbuilt references including DatabaseImmuneCellExpressionData, MonacoImmuneData, and NovershternHeamtopoieticData to improve its performance and domain knowledge on established markers of cell types. We additionally identified “cluster markers,” which were defined as DEGs, which were either more or less expressed within a chosen subset of cells/clusters compared with all other subsets/clusters within the Seurat object. This test was performed using “FindAllMarkers” function in Seurat with Wilcoxon rank sum test. Genes with >0.25 log-fold changes, at least 25% expressed in tested groups, and Bonferroni-corrected p-values <0.05 were regarded as cluster markers. The final cluster annotations were determined and verified based on the expression of cluster markers, legacy knowledge, and SingleR annotations. In the situations where multiple clusters were grouped, cluster markers were recomputed using the same methods, at the level of subsets.

### 4.6 Myeloid and Lymphoid Cell-Type Grouping

To discern the trends in cell-type changes and between cell-type interactions, we grouped the mononuclear cell types by origin: myeloid (MKs, basophils, mDCs, CD14^+^ CMs, CD16^+^ non-CMs (NCM), intermediate monocytes (ITM), and LDNs) and lymphoid (CD4^+^ and CD8^+^ T-cell subsets, MAIT cells, gamma-delta T(γδT), PBs, B cells, and NK cells). As per the most recent lympho-myeloid model of hematopoiesis (38), the DC phenotype exists on a spectrum from lymphoid (with plasmacytoid DCs most resembling lymphoid cells) to myelo-monocytic (with mDCs most resembling myeloid/monocytic cells). We subsequently annotate the plasmacytoid DCs as belonging to the lymphoid lineage (**Figure 1E**).

### 4.7 Subsampling

The original parent Seurat object was subsampled, for specific cellular compartments (e.g., PBs, LDN, monocytes, and NK cells) to increase cluster annotation granularity. That is, we divided the parent Seurat object into individual Seurat objects comprising cells from each compartment of interest. The integrated space within these subsampled cells was reclustered similar to the processing of the parent Seurat object/primary UMAP. Resolution for each subsampled space was chosen between 0.8 and 1.2. The resolution chosen for a cell type is directly indicated within the results. The cluster identities of the clusters identified within the subsampled spaces (minor clusters) were established using the same strategy as for the parent Seurat object. Some clusters were renamed based on cluster markers identified from SingleR annotation.

### 4.8 Establishing Cellular Heterogeneity of Subsampled Immune Cells

#### 4.8.1 Monocytes

Subsampling and reclustering the original 22 monocyte clusters (CD14^+^, CD16^+^, and ITM) resulted in a total of 19 clusters within the subsampled space, which was further grouped into 11 distinct monocytic subsets, as follows. Based on the expression of markers CD16, CD14, TNFRFA1, and TNFRFB2, clusters 3 and 13 were grouped into NCM (**Figure 2B**), while cluster 11 was identified as ITM based on the expression of markers CD14, CD16, HLA-DR, and TNFRFB2, and other known ITM gene markers including MARCO, APOBEC3A, MARCKSL1, and GBP4. ITMs showed significant enrichment (p.adj < 0.05) for members of the complement activation/signaling (C1QA, C1QB, C3AR1, C5AR1, and SERPINA1) and IFN-I response (**Supplementary Table S5**). Cluster 18 (expressing CD33 and ITGAM as cluster markers (p.adj < 0.05)) was annotated as CD33^+^ mMDSCs (27). mMDSCs were markedly absent within healthy samples. Both ITM and mMDSC had the highest cell abundances within severe disease (**Figure 2C**, **Supplementary Table S8, Supplementary Figure S3A**). Abundance signatures for mMDSCs were verified within an independent patient cohort and are consistent with our observations (**Supplementary Figure S3B**).

The remaining 15 clusters were divided into eight subsets of annotated CD14^+^ CM *via* SingleR annotation (**Figure 2D**). Several of the CM subsets were reminiscent of subtypes explored in previous publications and are not explored in detail within this manuscript including CM2 (cluster 4, MHC-II high), CM5 (clusters 1 and 12), and CM6 (cluster 5) (5, 6, 10). These subsets in addition to NCM had reduced abundances within COVID-19. Subset CM3 (clusters 7, 16, and 17) was found to have reduced expression of HLA-DR CD14, alarmins, and/or SELL and increased expression of tetraspanins (e.g., CD63 and CD53). The remaining CM subsets are described and analyzed within the main manuscript.

#### 4.8.2 Low-Density Neutrophils

Subsampling and reclustering “LDNs” from the parent Seurat object resulted in nine clusters (**Figure 3**, **Supplementary Figure S4**) grouped into three distinct subsets based on the expression patterns of established markers and cluster markers. Clusters 0, 1, 2, and 3 were identified as LDGs based on the mixed expression of activated mature neutrophil markers including CD11a/b/ITGAL/ITGAM, CD55, CD16, CD10/MME, and SELL and immature markers including CD16^high/low^ and CD10^low^ (33). Clusters 4, 6, and 8 were identified as PMN-MDSCs, based on the significant expression (p.adj < 0.05) of several known markers including ITGAM, LCN2, CAMP, MMP8, ARG1, S100A8, and S100A9 (37, 38) and were subsequently grouped together (**Figure 3E**). OLR1 (LOX1), a recently validated PMN-MDSC marker, was exclusively increased within this subset (p.adj < 0.05) (38) (**Figures 3E, F**). Clusters 5 and 7 expressed FUT4 (CD15), CD63, PRTN3, MPO, and ELANE reminiscent of a “proneutrophil” state (5).

#### 4.8.3 Natural Killer Cells

Subsetting and reclustering NK cells from the primary UMAP resulted in 23 distinct clusters (**Supplementary Figure S5A**). Three clusters (clusters 4, 6, and 19) showed significant enrichment of CD3 (CD3D/G) suggestive of an NKT (invariant NK-T) like population. Because most NK cells express CD7^+^, one CD7^−^ cluster (cluster 16) was excluded from further analysis (**Supplementary Figure S5B**) (64, 65). Reclustering and re-embedding of the Seurat object after processing from the original Seurat object resulted in 18 clusters (**Figure 4A**), grouped into seven distinct subsets based on previously published NK markers and considered for further analysis (**Figures 4B, C**, and **Supplementary Figure S5C**). NK4 (cluster 8), NK5 (cluster 14), and NK8 (clusters 2, 9, and 12) were identified as CD56^low^ subsets. Subsets NK3 (clusters 4–7, 10–11, and 17) and NK7 (clusters 0 and 1) largely lacked expression of CD56 (CD56^neg^) (**Supplementary Figure S5D**). Specifically, NK5, NK7, and NK8 represented activated/mature NK clusters based on the expression of cytotoxic markers including perforin (PRF1), granzymes (GZMA, GZMB, and GZMK), and remodeling markers including ACTB, ARPC3/4, CFL1, and CST7. NK3 showed an increased expression of inhibitory receptors including KIR2DL1/3, KIR3DL1/L2, KLRC2, IL32, and CD3E and cytolytic molecules including GZMH and CST7 and lower levels of FCER1G, in line with a profile of adaptive NK cells within increased cytotoxic potential (**Supplementary Table S16**). Two subsets NK6 (cluster 3, ~6.9% of total NK cells) and NK2 (clusters 16 and 15, ~6.5% of total NK cells) were characterized by increased expression of CD56. NK6 expressed surface proteins commonly attributed to CD56^bright^ population including CD44, SELL, and GZMK chemokines XCL1, XCL2, DUSP1, FOS, JUND, IL7R, and IL2RB (64).

#### 4.8.4 T Cells

Subsampling and reclustering the original 19 CD4/CD8T (and naïve) clusters comprising 128,589 cells resulted in a total of 22 clusters within the subsampled space. Using the commonly used subtyping markers, we observed four clusters (clusters 5, 19, 20, and 21) to be non-T specific (with a small population of CD3^+^ T cells) and were removed. Additionally, we observed that cluster 5 was majorly contributed by healthy samples, from only SS_C1, and was subsequently eliminated. The remaining 18 clusters (119,683 cells) were further grouped into eight distinct subsets (**Figures 5A, B**) using automatic annotation and verified based on the expression of commonly used T subtyping markers (**Supplementary Figures S6A, B**) (98). Specifically, based on the expression of naïve markers CCR7, SELL, and TCF7, seven CD4^+^ clusters (clusters 1, 4, 7, 11, 12, 14, and 15) were grouped into the naïve CD4T subset, while a single CD8^+^ cluster (cluster 10) was identified as the naïve CD8 T-cell subset. Likewise, based on the expression of granzymes (GZMA/B), PRF1, and NKG7, clusters 2, 3, and 9 were identified to be terminally differentiated effector (Teff) CD8T cells, while CD4^+^ cluster 3 was identified as Teff CD4T. Clusters 6 and 13 were grouped into Treg CD4T subset based on the expression of CTLA4, FOXP3, IL2R, and ICOS (**Supplementary Figure S6B**). Consistent with SingleR annotation, cluster 0 was identified as TFH (GATA3^+^), and cluster 17 was identified as memory T cells (Tmem, CD27^+^, IL7R^+^, CD28^+^, and FAS/CD95^+^). Studies have broadly identified increased exhaustion with increasing severity in the effector T-cell subset of COVID-19 patients (14). Recent research has additionally demonstrated a presence of clonally distinct hybrid memory T-cell subpopulation with an exhausted phenotype (GZMK^+^ and TOX^+^ with markers of exhaustion including PDCD1, MAF, and NFATC2), which is in contrast to the classical understanding that memory and exhausted T cells arise from segregated pathways (99). The CD8^+^ Tmem population identified in our study exhibits signatures consistent with this clonally distinct population, indicative of the transition of a subset of exhausted T cells to a memory stage in both severe and non-severe COVID-19 (cells in healthy = 361, non-severe = 656, and severe = 622).

#### 4.8.5 B Cells

We extracted the B-cell subsets (B lymphoblasts, naïve B cells, and non-switched memory B cells) from the original parent Seurat object (**Supplementary Figure S8A**) and identified the most significant remodeling occurring only within the naïve cell compartment. Subsampling and reclustering of naïve B cells resulted in 11 initial clusters (**Supplementary Figure 8B**). Out of the 11 initial clusters, we excluded two (clusters 6 and 7), as their most significantly upregulated cluster markers indicated potential erythroid and myeloid lineage cells and were not entirely consistent with naïve B cells. Of the remaining nine clusters (**Figure 6A**), we verified expression of surface markers consistent for naïve B cells (**Supplementary Figures S8C, D**). We further grouped these clusters into groups A–F, based on gross gene expression (via hierarchical clustering of scaled average gene expression values expressed in greater than 60% of all cells) in each cluster. In multiple instances, this unsupervised clustering method grouped together clusters that comprised similar proportions of healthy, non-severe, and severe cells (**Figure 6B**). In groups A (clusters 2 and 9) and B (clusters 5 and 8), non-severe cell numbers were much greater than in both healthy and severe cells. In group C (cluster 1), non-severe cells still dominate the cluster, but are followed by severe and then healthy cells. In group D (clusters 0 and 4), healthy and non-severe cells outnumber severe cells. Finally, in groups E (cluster 3) and F (cluster 10), severe cells are greater than non-severe and healthy cells.

Subsampling and reclustering PBs from the original parent object resulted in 10 clusters (**Supplementary Figure S8F**), of which six showed surface marker expression most characteristic of PBs (CD19^−^, MS4A1^−^, CD27^+^, and CD38^+^) and were retained for further analysis (**Supplementary Figure S8G**) (**Figures 6E–G**). The six clusters were grouped into four subsets PB1 (clusters 0 and 2), PB2 (clusters 3 and 6), PB3 (cluster 1), and PB4 (cluster 5) based on expression patterns among the top 60 cluster markers. For clarity, the expression of the top 20 genes from each cluster is shown in **Supplementary Figure S8H**. The clusters comprising subsets PB1, PB2, PB3, and PB4 and the number of healthy, non-severe, and severe cells in each subset are detailed in **Supplementary Figure S8I**. Since PBs from healthy patients comprised only about 5% of all PBs, we considered cells only from severe and non-severe patients in our analysis. In addition to an expanded PB population relative to non-severe patients, the PB population in severe patients was also more heterogeneous: no single subset dominated the PB response in severe disease, as multiple subsets were expanded; by contrast, subset PB1 dominated the PB response in non-severe disease.

### 4.9 Differential Gene Expression Analysis, Enrichment Analysis, and Visualization

Dotplots used within this manuscript were all generated using the “DotPlot” function of Seurat. The average expression presented within each dotplot is the scaled/standardized expression values as defined by Seurat. DGEA was performed with respect to either healthy or non-severe samples, depending on the context of analysis, using the “FindMarker” function within Seurat. The thresholds for calling DEGs were as discussed above. Heatmaps of the DEGs highlighting the fold changes across conditions of interest were performed using “pheatmap” library in R/BioC. Gene Ontology (GO) enrichment on the DEGs identified was performed using ClusterProfiler v.3.10.1 (100) or Enrichr (101). The top 10–15 categories of GO were utilized for visualization throughout the manuscript and **Supplementary Material**. Cluster profiler was also utilized for visualization of enrichment.

### 4.10 Transcription Factor Activity Characterization and Transcription Factor-Binding Enrichment

To characterize the transcriptional regulation of the altered genes programs within the subset, we queried TF activity *via* DoRothEA (Discriminate Regulon Expression Analysis) (102), which utilizes the viper activity inference algorithm against a curated list of TFs and its target expression levels to predict TF activity (regulon). DoRothEA regulons have been generated from various sources and evidence types, and we subsequently included sets with “A” or “B” confidence ratings. We visualized the top 20 genes ranked by DoRothEA for visualization. We also utilized ChEA3 (103) to rank TFs. In contrast to DoRothEA, which utilizes the entire expression matrix to derive putative TFs involved, ChEA3 predicts TF enrichment based on the overlap between given lists of DEGs or genes of interest, and TF targets assembled from a compendium of resource. ChEA3 utilizes a Fisher’s exact test, with a background size of 20,000, to compare the input gene set to the TF target-gene sets to establish significance. We utilized the top 10 highly ranked TFs defined *via* the “TopRank” metric for further visualization.

### 4.11 Independent Patient Cohort and Immunophenotyping of Myeloid-Derived Suppressor Cells, Mucosal-Associated Invariant T Cells, and Gamma-Delta T Cells

*Human Subjects*: Blood samples from healthy subjects were enrolled and tested under Plexision IRB-approved protocol 6774. COVID-19 patients were enrolled under IRB-approved protocol #1551551 from Edinburg, TX. Patients with COVID-19 infection are categorized into three groups, COVID-19 patients who required no hospitalization (Mild), those who required hospitalization with no mechanical ventilation (Hospitalized), and those who were hospitalized and required ventilation for oxygen requirements (Severe). Patient cohort demographics and severity details are presented within **Supplementary Table S19**.

To provide independent validation of expansion of certain immune cell populations in severe and non-severe COVID-19 and healthy controls, we performed flow cytometric analyses on PBMCs from the above independent patient cohort. Cells from PBMCs were labeled with fluorochrome-labeled antibodies to characterize mMDSC and PMN-MDSC subsets. The respective phenotypes were CD14^+^HLA-DR^−^ and CD14-CD15^+^CD11b^+^. MAIT cells were gated based on CD3^+^ TCRγδ-TCRVα7.2^+^CD161^+^ cells, and γδT cells were identified based on CD3^+^ TCRγδ^+^ cells. Antibodies from BioLegend (San Diego, CA) and BD Biosciences (San Jose, CA) were utilized.

### 4.12 RNA-Binding Proteins

The current knowledgebase of all RBPs was downloaded from RBPBase (104) containing 4,257 RBPs within humans. Of these, we considered only those RBPs that are present in the top 25% of the hits_Hs (>6), bringing down the list to 1,031 high-confidence RBPs. Consensus genes that overlapped with this list are provided in **Supplementary Figure S2C**.

## Data Availability Statement

The original contributions presented in the study are included in the article/**Supplementary Material**. Further inquiries can be directed to the corresponding author.

## Ethics Statement

The studies involving human participants were reviewed and approved by IRB-approved protocol #6774 and IRB-approved protocol #1551551. The patients/participants provided their written informed consent to participate in this study.

## Author Contributions

Conceptualization: KM and SS. Methodology: KM and SS. Investigation: KM and PN (transcriptomics) and CA and RS (immunophenotyping). Visualization: KM and PN (transcriptomics) and CA and RS (immunophenotyping). Independent patient cohort acquisition: SR, JA, and MB-G. Funding acquisition: SS. Supervision: SS. Writing—original draft: KM. Writing—review and editing: KM, PN, CA, RS, and SS. All authors contributed to the article and approved the submitted version.

## Funding

National Institutes of Health grant R01 LM012595 (SS); National Institutes of Health grant U19 AI090023 (SS); National Institutes of Health grant R01 HL108735 (SS); National Institutes of Health grant OT2 1 OD030544 (SS); and Joan and Irwin Jacobs Endowment (SS).

## Conflict of Interest

Phenotyping of the independent cohort was performed at Plexision, a University of Pittsburgh spinoff in which RS and the University hold equity. RS and CA are currently employed at Plexision.

The remaining authors declare that the research was conducted in the absence of any commercial or financial relationships that could be construed as a potential conflict of interest.

## Publisher’s Note

All claims expressed in this article are solely those of the authors and do not necessarily represent those of their affiliated organizations, or those of the publisher, the editors and the reviewers. Any product that may be evaluated in this article, or claim that may be made by its manufacturer, is not guaranteed or endorsed by the publisher.

## References

[1] Lavine JS Bjornstad ON Antia R. Immunological Characteristics Govern the Transition of COVID-19 to Endemicity. Science (2021) 371(6530):741–5. 10.1126/science.abe6522 PMC7932103 33436525

[2] Mukund K Mathee K Subramaniam S. Plasmin Cascade Mediates Thrombotic Events in SARS-CoV-2 Infection *via* Complement and Platelet-Activating Systems. IEEE Open J Eng Med Biol (2020) 1:220–7. doi: 10.1109/OJEMB.2020.3014798 PMC8527892 34786557

[3] Blanco-Melo D Nilsson-Payant B Liu WC Uhl S Hoagland D Møller R. Imbalanced Host Response to SARS-CoV-2 Drives Development of COVID-19. Cell (2020) 181(5):1036–45. doi: 10.1016/j.cell.2020.04.026 PMC7227586 32416070

[4] Lee JS Park S Jeong HW Ahn JY Choi SJ Lee H. Immunophenotyping of COVID-19 and Influenza Highlights the Role of Type I Interferons in Development of Severe COVID-19. Sci Immunol (2020) 5(49):eabd1554. doi: 10.1126/sciimmunol.abd1554 32651212 PMC7402635

[5] Schulte-Schrepping J Reusch N Paclik D Baßler K Schlickeiser S Zhang B. Severe COVID-19 Is Marked by a Dysregulated Myeloid Cell Compartment. Cell (2020) 182:1419–40. doi: 10.1016/j.cell.2020.08.001 PMC7405822 32810438

[6] Bernardes JP Mishra N Tran F Bahmer T Best L Blase JI. Longitudinal Multi-Omics Analyses Identify Responses of Megakaryocytes, Erythroid Cells, and Plasmablasts as Hallmarks of Severe COVID-19. Immunity (2020) 53:1296–314. doi: 10.1016/j.immuni.2020.11.017 PMC7689306 33296687

[7] Mathew D Giles JR Baxter AE Oldridge DA Greenplate AR Wu JE. Deep Immune Profiling of COVID-19 Patients Reveals Distinct Immunotypes With Therapeutic Implications. Science (2020) 369(6508):eabc8511. 32669297 10.1126/science.abc8511 PMC7402624

[8] Wang T Du Z Zhu F Cao Z An Y Gao Y. Comorbidities and Multi-Organ Injuries in the Treatment of COVID-19. Lancet (2020) 395:e52. doi: 10.1016/S0140-6736(20)30558-4 32171074 PMC7270177

[9] Osman M Faridi RM Sligl W Shabani-Rad M-T Dharmani-Khan P Parker A. Impaired Natural Killer Cell Counts and Cytolytic Activity in Patients With Severe COVID-19. Blood Adv (2020) 4:5035–9. doi: 10.1182/bloodadvances.2020002650 PMC7594380 33075136

[10] Wilk AJ Rustagi A Zhao NQ Roque J Martínez-Colón GJ McKechnie JL. A Single-Cell Atlas of the Peripheral Immune Response in Patients With Severe COVID-19. Nat Med (2020) 26(7):1070–6. doi: 10.1038/s41591-020-0944-y PMC7382903 32514174

[11] Kuri-Cervantes L Pampena MB Meng W Rosenfeld AM Ittner CA Weisman AR. Comprehensive Mapping of Immune Perturbations Associated With Severe COVID-19. Sci Immunol (2020) 5(49):eabd7114. doi: 10.1126/sciimmunol.abd7114 32669287 PMC7402634

[12] Yao C Bora SA Parimon T Zaman T Friedman OA Palatinus JA. Cell-Type-Specific Immune Dysregulation in Severely Ill COVID-19 Patients. Cell Rep (2021) 34:108590. doi: 10.1016/j.celrep.2020.108590 33357411 PMC7744012

[13] Su Y Chen D Yuan D Lausted C Choi J Dai CL. Multi-Omics Resolves a Sharp Disease-State Shift Between Mild and Moderate COVID-19. Cell (2020) 183:1479–95. doi: 10.1016/j.cell.2020.10.037 PMC7598382 33171100

[14] Diao B Wang C Tan Y Chen X Liu Y Ning L. Reduction and Functional Exhaustion of T Cells in Patients With Coronavirus Disease 2019 (COVID-19). Front Immunol (2020) 11:827. doi: 10.3389/fimmu.2020.00827 32425950 PMC7205903

[15] Hadjadj J Yatim N Barnabei L Corneau A Boussier J Smith N. Impaired Type I Interferon Activity and Inflammatory Responses in Severe COVID-19 Patients. Science (2020) 369:718–24. doi: 10.1126/science.abc6027 PMC7402632 32661059

[16] Ren X Wen W Fan X Hou W Su B Cai P. COVID-19 Immune Features Revealed by a Large-Scale Single-Cell Transcriptome Atlas. Cell (2021) 184:1895–913. doi: 10.1016/j.cell.2021.01.053 PMC7857060 33657410

[17] Sosa-Hernández VA Torres-Ruíz J Cervantes-Díaz R Romero-Ramírez S Páez-Franco JC Meza-Sánchez DE. B Cell Subsets as Severity-Associated Signatures in COVID-19 Patients. Front Immunol (2020) 11:3244. doi: 10.3389/fimmu.2020.611004 PMC7744304 33343585

[18] Dan JM Mateus J Kato Y Hastie KM Yu ED Faliti CE. Immunological Memory to SARS-CoV-2 Assessed for Up to 8 Months After Infection. Science (2021) 371(6529):464–5. doi: 10.1126/science.abf4063 PMC7919858 33408181

[19] Peng Y Mentzer AJ Liu G Yao X Yin Z Dong D. Broad and Strong Memory CD4+ and CD8+ T Cells Induced by SARS-CoV-2 in UK Convalescent Individuals Following COVID-19. Nat Immunol (2020) 21:1336–45. doi: 10.1038/s41590-020-0782-6 PMC7611020 32887977

[20] Arunachalam PS Wimmers F Mok CKP Perera RA Scott M Hagan T. Systems Biological Assessment of Immunity to Mild Versus Severe COVID-19 Infection in Humans. Science (2020) 369:1210–20. doi: 10.1126/science.abc6261 PMC7665312 32788292

[21] Stephenson E Reynolds G Botting RA Calero-Nieto FJ Morgan MD Tuong ZK. Single-Cell Multi-Omics Analysis of the Immune Response in COVID-19. Nat Med (2021) 27:904–16. doi: 10.1038/s41591-021-01329-2 PMC8121667 33879890

[22] Lamers MM van den Hoogen BG Haagmans BL. Adar1: “Editor-in-Chief” of Cytoplasmic Innate Immunity. Front Immunol (2019) 10:1763. doi: 10.3389/fimmu.2019.01763 31404141 PMC6669771

[23] Vavougios GD. SARS-CoV-2 Dysregulation of PTBP1 and YWHAE/Z Gene Expression: A Primer of Neurodegeneration. Med Hypotheses (2020) 144:110212. doi: 10.1016/j.mehy.2020.110212 33254518 PMC7448818

[24] Bonny TS Patel EU Zhu X Bloch EM Grabowski MK Abraham AG. Cytokine and Chemokine Levels in Coronavirus Disease 2019 Convalescent Plasma. Open Forum Infect Dis (2020) 8(2):ofaa574. doi: 10.1093/ofid/ofaa574 33553467 PMC7717355

[25] Akgun E Tuzuner MB Sahin B Kilercik M Kulah C Cakiroglu HN. Proteins Associated With Neutrophil Degranulation Are Upregulated in Nasopharyngeal Swabs From SARS-CoV-2 Patients. PloS One (2020) 15:e0240012. doi: 10.1371/journal.pone.0240012 33079950 PMC7575075

[26] Merah-Mourah F Cohen S Charron D Mooney N Haziot A. Identification of Novel Human Monocyte Subsets and Evidence for Phenotypic Groups Defined by Interindividual Variations of Expression of Adhesion Molecules. Sci Rep (2020) 10:1–16. doi: 10.1038/s41598-020-61022-1 32157175 PMC7064612

[27] Canè S Ugel S Trovato R Marigo I De Sanctis F Sartoris S. The Endless Saga of Monocyte Diversity. Front Immunol (2019) 10:1786. doi: 10.3389/fimmu.2019.01786 31447834 PMC6691342

[28] Reyes M Filbin MR Bhattacharyya RP Sonny A Mehta A Billman K. Plasma From Patients With Bacterial Sepsis or Severe COVID-19 Induces Production of Suppressive Myeloid Cells From Human Hematopoietic Progenitor Cells *In Vitro* . Sci Trans Med (2021) 13(598):eabe9599. doi: 10.1126/scitranslmed.abe9599 PMC8432955 34103408

[29] Guo C Li B Ma H Wang X Cai P Yu Q. Single-Cell Analysis of Two Severe COVID-19 Patients Reveals a Monocyte-Associated and Tocilizumab-Responding Cytokine Storm. Nat Commun (2020) 11:1–11. doi: 10.1038/s41467-020-17834-w 32764665 PMC7413381

[30] Delano MJ Scumpia PO Weinstein JS Coco D Nagaraj S Kelly-Scumpia KM. MyD88-Dependent Expansion of an Immature GR-1+ CD11b+ Population Induces T Cell Suppression and Th2 Polarization in Sepsis. J Exp Med (2007) 204:1463–74. doi: 10.1084/jem.20062602 PMC2118626 17548519

[31] Shalova IN Lim JY Chittezhath M Zinkernagel AS Beasley F Hernández-Jiménez E. Human Monocytes Undergo Functional Re-Programming During Sepsis Mediated by Hypoxia-Inducible Factor-1α. Immunity (2015) 42:484–98. doi: 10.1016/j.immuni.2015.02.001 25746953

[32] Cai B Wu J Yu X Su X Wang R-F. FOSL1 Inhibits Type I Interferon Responses to Malaria and Viral Infections by Blocking TBK1 and TRAF3/TRIF Interactions. MBio (2017) 8(1):e02161–16. doi: 10.1128/mBio.02161-16 PMC5210502 28049150

[33] Wang J Sun D Wang Y Ren F Pang S Wang D. FOSL2 Positively Regulates TGF-β1 Signalling in Non-Small Cell Lung Cancer. PloS One (2014) 9:e112150. doi: 10.1371/journal.pone.0112150 25375657 PMC4223012

[34] Grunwell JR Yeligar SM Stephenson S Du Ping X Gauthier TW Fitzpatrick AM. TGF-β1 Suppresses the Type I IFN Response and Induces Mitochondrial Dysfunction in Alveolar Macrophages. J Immunol (2018) 200:2115–28. doi: 10.4049/jimmunol.1701325 PMC5928790 29427413

[35] Golden JB Groft SG Squeri MV Debanne SM Ward NL McCormick TS. Chronic Psoriatic Skin Inflammation Leads to Increased Monocyte Adhesion and Aggregation. J Immunol (2015) 195:2006–18. doi: 10.4049/jimmunol.1402307 PMC4686256 26223654

[36] Saeed Z Rowan A Greiller C Taylor GP Pollock KM. Enhanced T-Cell Maturation and Monocyte Aggregation Are Features of Cellular Inflammation in Human T-Lymphotropic Virus Type 1–Associated Myelopathy. Clin Infect Dis (2020) 70:1326–35. doi: 10.1093/cid/ciz369 31063543

[37] Hottz ED Azevedo-Quintanilha IG Palhinha L Teixeira L Barreto EA Pão CR. Platelet Activation and Platelet-Monocyte Aggregate Formation Trigger Tissue Factor Expression in Patients With Severe COVID-19. Blood J Am Soc Hematol (2020) 136:1330–41. doi: 10.1182/blood.2020007252 PMC7483437 32678428

[38] Collin M Bigley V. Human Dendritic Cell Subsets: An Update. Immunology (2018) 154:3–20. doi: 10.1111/imm.12888 29313948 PMC5904714

[39] Sánchez-Cerrillo I Landete P Aldave B Sánchez-Alonso S Sánchez-Azofra A Marcos-Jiménez A. COVID-19 Severity Associates With Pulmonary Redistribution of CD1c+ DCs and Inflammatory Transitional and Nonclassical Monocytes. J Clin Invest (2020) 130(12). doi: 10.1172/JCI140335 PMC7685723 32784290

[40] Li Z Ju X Silveira PA Abadir E Hsu W-H Hart DN. CD83: Activation Marker for Antigen Presenting Cells and Its Therapeutic Potential. Front Immunol (2019) 10:1312. doi: 10.3389/fimmu.2019.01312 31231400 PMC6568190

[41] Saini A Mahajan S Gupta P. Nuclear Receptor Expression Atlas in BMDCs: Nr4a2 Restricts Immunogenicity of BMDCs and Impedes EAE. Eur J Immunol (2016) 46:1842–53. doi: 10.1002/eji.201546229 27184189

[42] Shin J-S Greer AM. The Role of Fcϵri Expressed in Dendritic Cells and Monocytes. Cell Mol Life Sci (2015) 72:2349–60. doi: 10.1007/s00018-015-1870-x PMC4479177 25715742

[43] Nikitina E Larionova I Choinzonov E Kzhyshkowska J. Monocytes and Macrophages as Viral Targets and Reservoirs. Int J Mol Sci (2018) 19:2821. doi: 10.3390/ijms19092821 PMC6163364 30231586

[44] Gicquel T Robert S Loyer P Victoni T Bodin A Ribault C. Ilr1β Production is Dependent on the Activation of Purinergic Receptors and NLRP3 Pathway in Human Macrophages. FASEB J (2015) 29:4162–73. doi: 10.1096/fj.14-267393 26116704

[45] Hassani M Hellebrekers P Chen N van Aalst C Bongers S Hietbrink F. On the Origin of Low-Density Neutrophils. J Leukocyte Biol (2020) 107:809–18. doi: 10.1002/JLB.5HR0120-459R PMC7318192 32170882

[46] Silvestre-Roig C Fridlender ZG Glogauer M Scapini P. Neutrophil Diversity in Health and Disease. Trends Immunol (2019) 40:565–83. doi: 10.1016/j.it.2019.04.012 PMC7185435 31160207

[47] Zhu YP Padgett L Dinh HQ Marcovecchio P Blatchley A Wu R. Identification of an Early Unipotent Neutrophil Progenitor With Pro-Tumoral Activity in Mouse and Human Bone Marrow. Cell Rep (2018) 24:2329–41. doi: 10.1016/j.celrep.2018.07.097 PMC6542273 30157427

[48] Alshetaiwi H Pervolarakis N McIntyre LL Ma D Nguyen Q Rath JA. Defining the Emergence of Myeloid-Derived Suppressor Cells in Breast Cancer Using Single-Cell Transcriptomics. Sci Immunol (2020) 5(44). doi: 10.1126/sciimmunol.aay6017 PMC7219211 32086381

[49] Skokowa J Welte K. LEF-1 is a Decisive Transcription Factor in Neutrophil Granulopoiesis. Ann NY Acad Sci (2007) 1106:143–51. doi: 10.1196/annals.1392.012 17360796

[50] Scapini P Marini O Tecchio C Cassatella MA. Human Neutrophils in the Saga of Cellular Heterogeneity: Insights and Open Questions. Immunol Rev (2016) 273:48–60. doi: 10.1111/imr.12448 27558327

[51] Karlsson T Glogauer M Ellen RP Loitto V Magnusson K Magalhaes MA. Aquaporin 9 Phosphorylation Mediates Membrane Localization and Neutrophil Polarization. J Leukocyte Biol (2011) 90:963–73. doi: 10.1189/jlb.0910540 21873454

[52] Rocha BC Marques PE de Souza Leoratti FM Junqueira C Pereira DB do Valle Antonelli LR. Type I Interferon Transcriptional Signature in Neutrophils and Low-Density Granulocytes Are Associated With Tissue Damage in Malaria. Cell Rep (2015) 13:2829–41. doi: 10.1016/j.celrep.2015.11.055 PMC4698035 26711347

[53] Rahman S Sagar D Hanna RN Lightfoot YL Mistry P Smith CK. Low-Density Granulocytes Activate T Cells and Demonstrate a non-Suppressive Role in Systemic Lupus Erythematosus. Ann Rheum Dis (2019) 78:957–66. doi: 10.1136/annrheumdis-2018-214620 PMC6585283 31040119

[54] Rios-Santos F Alves-Filho JC Souto FO Spiller F Freitas A Lotufo CMC. Down-Regulation of CXCR2 on Neutrophils in Severe Sepsis is Mediated by Inducible Nitric Oxide Synthase–Derived Nitric Oxide. Am J Respir Crit Care Med (2007) 175:490–7. doi: 10.1164/rccm.200601-103OC 17138957

[55] Aarts CE Hiemstra IH Béguin EP Hoogendijk AJ Bouchmal S van Houdt M. Activated Neutrophils Exert Myeloid-Derived Suppressor Cell Activity Damaging T Cells Beyond Repair. Blood Adv (2019) 3:3562–74. doi: 10.1182/bloodadvances.2019031609 PMC6880908 31738831

[56] Fernando V Zheng X Walia Y Sharma V Letson J Furuta S. S-Nitrosylation: An Emerging Paradigm of Redox Signaling. Antioxidants (2019) 8:404. doi: 10.3390/antiox8090404 PMC6769533 31533268

[57] Campbell RA Schwertz H Hottz ED Rowley JW Manne BK Washington AV. Human Megakaryocytes Possess Intrinsic Antiviral Immunity Through Regulated Induction of IFITM3. Blood (2019) 133:2013–26. doi: 10.1182/blood-2018-09-873984 PMC6509546 30723081

[58] Valdivia-Mazeyra MF Salas C Nieves-Alonso JM Martín-Fragueiro L Bárcena C Muñoz-Hernández P. Increased Number of Pulmonary Megakaryocytes in COVID-19 Patients With Diffuse Alveolar Damage: An Autopsy Study With Clinical Correlation and Review of the Literature. Virchows Archiv (2021) 478:487–96. doi: 10.1007/s00428-020-02926-1 PMC7483503 32915265

[59] Li Q Yang M Xia R Xia L Zhang L. Elevated Expression of IL-12 and IL-23 in Patients With Primary Immune Thrombocytopenia. Platelets (2015) 26:453–8. doi: 10.3109/09537104.2014.934217 25025295

[60] Lippi G Plebani M Henry BM. Thrombocytopenia Is Associated With Severe Coronavirus Disease 2019 (COVID-19) Infections: A Meta-Analysis. Clinica Chimica Acta (2020) 506:145–8. doi: 10.1016/j.cca.2020.03.022 PMC7102663 32178975

[61] Cunin P Nigrovic PA. Megakaryocyte Emperipolesis: A New Frontier in Cell-in-Cell Interaction. Platelets (2020) 31:700–6. doi: 10.1080/09537104.2019.1693035 PMC7239726 31752579

[62] Centurione L Di Baldassarre A Zingariello M Bosco D Gatta V Rana RA. Increased and Pathologic Emperipolesis of Neutrophils Within Megakaryocytes Associated With Marrow Fibrosis in GATA-1low Mice. Blood (2004) 104:3573–80. doi: 10.1182/blood-2004-01-0193 15292068

[63] Maucourant C Filipovic I Ponzetta A Aleman S Cornillet M Hertwig L. Natural Killer Cell Immunotypes Related to COVID-19 Disease Severity. Sci Immunol (2020) 5(50):eabd6832. doi: 10.1126/sciimmunol.abd6832 32826343 PMC7665314

[64] Yang C Siebert JR Burns R Gerbec ZJ Bonacci B Rymaszewski A. Heterogeneity of Human Bone Marrow and Blood Natural Killer Cells Defined by Single-Cell Transcriptome. Nat Commun (2019) 10:1–16. doi: 10.1038/s41467-019-11947-7 31477722 PMC6718415

[65] Smith SL Kennedy PR Stacey KB Worboys JD Yarwood A Seo S. Diversity of Peripheral Blood Human NK Cells Identified by Single-Cell RNA Sequencing. Blood Adv (2020) 4:1388–406. doi: 10.1182/bloodadvances.2019000699 PMC7160259 32271902

[66] Poli A Michel T Thérésine M Andrès E Hentges F Zimmer J. CD56bright Natural Killer (NK) Cells: An Important NK Cell Subset. Immunology (2009) 126:458–65. doi: 10.1111/j.1365-2567.2008.03027.x PMC2673358 19278419

[67] Orrantia A Terrén I Izquierdo-Lafuente A Alonso-Cabrera JA Sandá V Vitallé J. A NKp80-Based Identification Strategy Reveals That CD56neg NK Cells are Not Completely Dysfunctional in Health and Disease. Iscience (2020) 23:101298. doi: 10.1016/j.isci.2020.101298 32622268 PMC7334412

[68] Forconi CS Oduor CI Oluoch PO Ong’echa JM Münz C Bailey JA. A New Hope for CD56negCD16pos NK Cells as Unconventional Cytotoxic Mediators: An Adaptation to Chronic Diseases. Front Cell Infect Microbiol (2020) 10:162. doi: 10.3389/fcimb.2020.00162 32373555 PMC7186373

[69] Zitvogel L Kroemer G. Targeting Foxp1 for Reinstating Anticancer Immunosurveillance. Immunity (2014) 41:345–7. doi: 10.1016/j.immuni.2014.09.001 25238089

[70] Madera S Rapp M Firth MA Beilke JN Lanier LL Sun JC. Type I IFN Promotes NK Cell Expansion During Viral Infection by Protecting NK Cells Against Fratricide. J Exp Med (2016) 213:225–33. doi: 10.1084/jem.20150712 PMC4749923 26755706

[71] De Biasi S Meschiari M Gibellini L Bellinazzi C Borella R Fidanza L. Marked T Cell Activation, Senescence, Exhaustion and Skewing Towards TH17 in Patients With COVID-19 Pneumonia. Nat Commun (2020) 11:1–17. doi: 10.1038/s41467-020-17292-4 32632085 PMC7338513

[72] Sakaguchi S Yamaguchi T Nomura T Ono M. Regulatory T Cells and Immune Tolerance. Cell (2008) 133:775–87. doi: 10.1016/j.cell.2008.05.009 18510923

[73] Björkström NK Gonzalez VD Malmberg K-J Falconer K Alaeus A Nowak G. Elevated Numbers of Fcγriiia+ (CD16+) Effector CD8 T Cells With NK Cell-Like Function in Chronic Hepatitis C Virus Infection. J Immunol (2008) 181:4219–28. doi: 10.4049/jimmunol.181.6.4219 18768879

[74] Martos SN Campbell MR Lozoya OA Wang X Bennett BD Thompson IJ. Single-Cell Analyses Identify Dysfunctional CD16+ CD8 T Cells in Smokers. Cell Rep Med (2020) 1:100054. doi: 10.1016/j.xcrm.2020.100054 33163982 PMC7644053

[75] Krummel MF Mahale JN Uhl LF Hardison EA Mujal AM Mazet JM. Paracrine Costimulation of IFN-γ Signaling by Integrins Modulates CD8 T Cell Differentiation. Proc Natl Acad Sci (2018) 115:11585–90. doi: 10.1073/pnas.1804556115 PMC6233119 30348790

[76] Provine NM Klenerman P. MAIT Cells in Health and Disease. Annu Rev Immunol (2020) 38:203–28. doi: 10.1146/annurev-immunol-080719-015428 31986071

[77] Li Y Zhang Z Yang L Lian X Xie Y Li S. The MERS-CoV Receptor DPP4 as a Candidate Binding Target of the SARS-CoV-2 Spike. Iscience (2020) 23:101160. doi: 10.1016/j.isci.2020.101160 32405622 PMC7219414

[78] Parrot T Gorin J-B Ponzetta A Maleki KT Kammann T Emgård J. MAIT Cell Activation and Dynamics Associated With COVID-19 Disease Severity. Sci Immunol (2020) 5(51):eabe1670. doi: 10.1101/2020.08.27.20182550 32989174 PMC7857393

[79] De Biasi S Lo Tartaro D Meschiari M Gibellini L Bellinazzi C Borella R. Expansion of Plasmablasts and Loss of Memory B Cells in Peripheral Blood From COVID-19 Patients With Pneumonia. Eur J Immunol (2020) 50:1283–94. doi: 10.1002/eji.202048838 32910469

[80] Newman R Ahlfors H Saveliev A Galloway A Hodson DJ Williams R. Maintenance of the Marginal-Zone B Cell Compartment Specifically Requires the RNA-Binding Protein ZFP36L1. Nat Immunol (2017) 18:683–93. doi: 10.1038/ni.3724 PMC5438597 28394372

[81] Engelmann A Speidel D Bornkamm G Deppert W Stocking C. Gadd45β is a Pro-Survival Factor Associated With Stress-Resistant Tumors. Oncogene (2008) 27:1429–38. doi: 10.1038/sj.onc.1210772 17891184

[82] Ise W Fujii K Shiroguchi K Ito A Kometani K Takeda K. T Follicular Helper Cell-Germinal Center B Cell Interaction Strength Regulates Entry Into Plasma Cell or Recycling Germinal Center Cell Fate. Immunity (2018) 48:702–15. doi: 10.1016/j.immuni.2018.03.027 29669250

[83] Scharer CD Patterson DG Mi T Price MJ Hicks SL Boss JM. Antibody-Secreting Cell Destiny Emerges During the Initial Stages of B-Cell Activation. Nat Commun (2020) 11:1–14. doi: 10.1038/s41467-020-17798-x 32778653 PMC7417592

[84] Kunkel EJ Kim CH Lazarus NH Vierra MA Soler D Bowman EP. CCR10 Expression is a Common Feature of Circulating and Mucosal Epithelial Tissue IgA Ab-Secreting Cells. J Clin Invest (2003) 111:1001–10. doi: 10.1172/JCI17244 PMC152588 12671049

[85] Cheng Q Khodadadi L Taddeo A Klotsche J F. Hoyer B Radbruch A. CXCR4–CXCL12 Interaction is Important for Plasma Cell Homing and Survival in NZB/W Mice. Eur J Immunol (2018) 48:1020–9. doi: 10.1002/eji.201747023 29427452

[86] Tarte K Zhan F De Vos J Klein B Shaughnessy J. Gene Expression Profiling of Plasma Cells and Plasmablasts: Toward a Better Understanding of the Late Stages of B-Cell Differentiation. Blood (2003) 102:592–600. doi: 10.1182/blood-2002-10-3161 12663452

[87] Zhu H Bhatt B Sivaprakasam S Cai Y Liu S Kodeboyina SK. Ufbp1 Promotes Plasma Cell Development and ER Expansion by Modulating Distinct Branches of UPR. Nat Commun (2019) 10:1–15. doi: 10.1038/s41467-019-08908-5 30842412 PMC6403283

[88] Lee JS Shin E-C. The Type I Interferon Response in COVID-19: Implications for Treatment. Nat Rev Immunol (2020) 20:585–6. doi: 10.1038/s41577-020-00429-3 PMC8824445 32788708

[89] Guo Q Zhao Y Li J Liu J Yang X Guo X. Induction of Alarmin S100A8/A9 Mediates Activation of Aberrant Neutrophils in the Pathogenesis of COVID-19. Cell Host Microbe (2021) 29:222–35. doi: 10.1016/j.chom.2020.12.016 PMC7762710 33388094

[90] Schönrich G Raftery MJ Samstag Y. Devilishly Radical NETwork in COVID-19: Oxidative Stress, Neutrophil Extracellular Traps (NETs), and T Cell Suppression. Adv Biol Regul (2020) 77:100741. doi: 10.1016/j.jbior.2020.100741 32773102 PMC7334659

[91] Pasquevich KA Bieber K Günter M Grauer M Pötz O Schleicher U. Innate Immune System Favors Emergency Monopoiesis at the Expense of DC-Differentiation to Control Systemic Bacterial Infection in Mice. Eur J Immunol (2015) 45:2821–33. doi: 10.1002/eji.201545530 26138432

[92] Zuo Y Zuo M Yalavarthi S Gockman K Madison JA Shi H. Neutrophil Extracellular Traps and Thrombosis in COVID-19. J Thromb Thromb (2020) 51:446–53. doi: 10.1007/s11239-020-02324-z PMC7642240 33151461

[93] Morrissey SM Geller AE Hu X Tieri D Cooke EA Ding C. A Specific Low-Density Neutrophil Population Correlates With Hypercoagulation and Disease Severity in Hospitalized COVID-19 Patients. JCI Insight (2021) 6(9). doi: 10.1172/jci.insight.148435 PMC8262329 33986193

[94] Carissimo G Xu W Kwok I Abdad MY Chan Y-H Fong S-W. Whole Blood Immunophenotyping Uncovers Immature Neutrophil-to-VD2 T-Cell Ratio as an Early Marker for Severe COVID-19. Nat Commun (2020) 11:1–12. doi: 10.1038/s41467-020-19080-6 33067472 PMC7568554

[95] Costantini C Cassatella MA. The Defensive Alliance Between Neutrophils and NK Cells as a Novel Arm of Innate Immunity. J Leukocyte Biol (2011) 89:221–33. doi: 10.1189/jlb.0510250 20682626

[96] Schneider M Hannaway RF Lamichhane R de la Harpe SM Tyndall JD Vernall AJ. Neutrophils Suppress Mucosal-Associated Invariant T Cells in Humans. Eur J Immunol (2020) 50:643–55. doi: 10.1002/eji.201948394 31944287

[97] Stuart T Butler A Hoffman P Hafemeister C Papalexi E Mauck WM III. Comprehensive Integration of Single-Cell Data. Cell (2019) 177:1888–902. doi: 10.1016/j.cell.2019.05.031 PMC6687398 31178118

[98] Szabo PA Levitin HM Miron M Snyder ME Senda T Yuan J. Single-Cell Transcriptomics of Human T Cells Reveals Tissue and Activation Signatures in Health and Disease. Nat Commun (2019) 10:1–16. doi: 10.1038/s41467-019-12464-3 31624246 PMC6797728

[99] Galletti G De Simone G Mazza EM Puccio S Mezzanotte C Bi TM. Two Subsets of Stem-Like CD8+ Memory T Cell Progenitors With Distinct Fate Commitments in Humans. Nat Immunol (2020) 21:1552–62. doi: 10.1038/s41590-020-0791-5 PMC7610790 33046887

[100] Yu G Wang L-G Han Y He Q-Y. Clusterprofiler: An R Package for Comparing Biological Themes Among Gene Clusters. OMICS (2012) 16:284–7. doi: 10.1089/omi.2011.0118 PMC3339379 22455463

[101] Kuleshov MV Jones MR Rouillard AD Fernandez NF Duan Q Wang Z. Enrichr: A Comprehensive Gene Set Enrichment Analysis Web Server 2016 Update. Nucleic Acids Res (2016) 44:W90–7. doi: 10.1093/nar/gkw377 PMC4987924 27141961

[102] Holland CH Tanevski J Perales-Patón J Gleixner J Kumar MP Mereu E. Robustness and Applicability of Transcription Factor and Pathway Analysis Tools on Single-Cell RNA-Seq Data. Genome Biol (2020) 21:36. doi: 10.1186/s13059-020-1949-z 32051003 PMC7017576

[103] Keenan AB Torre D Lachmann A Leong AK Wojciechowicz ML Utti V. ChEA3: Transcription Factor Enrichment Analysis by Orthogonal Omics Integration. Nucleic Acids Res (2019) 47:W212–24. doi: 10.1093/nar/gkz446 PMC6602523 31114921

[104] Gebauer F Schwarzl T Valcárcel J Hentze MW. RNA-Binding Proteins in Human Genetic Disease. Nat Rev Genet (2020) 22:185–98. doi: 10.1038/s41576-020-00302-y 33235359

