# Supplementary material for: Immune Response in Severe and Non-Severe Coronavirus Disease 2019 (COVID-19) Infection: A Mechanistic Landscape
Source: Front Immunol. 2021 Oct 13;12:738073. doi: 10.3389/fimmu.2021.738073 (PMC8548832; doi:10.3389/fimmu.2021.738073)
Supplement: Supplementary file 1 [file DataSheet_1.docx]

Supplementary Material

Contd. On page 2


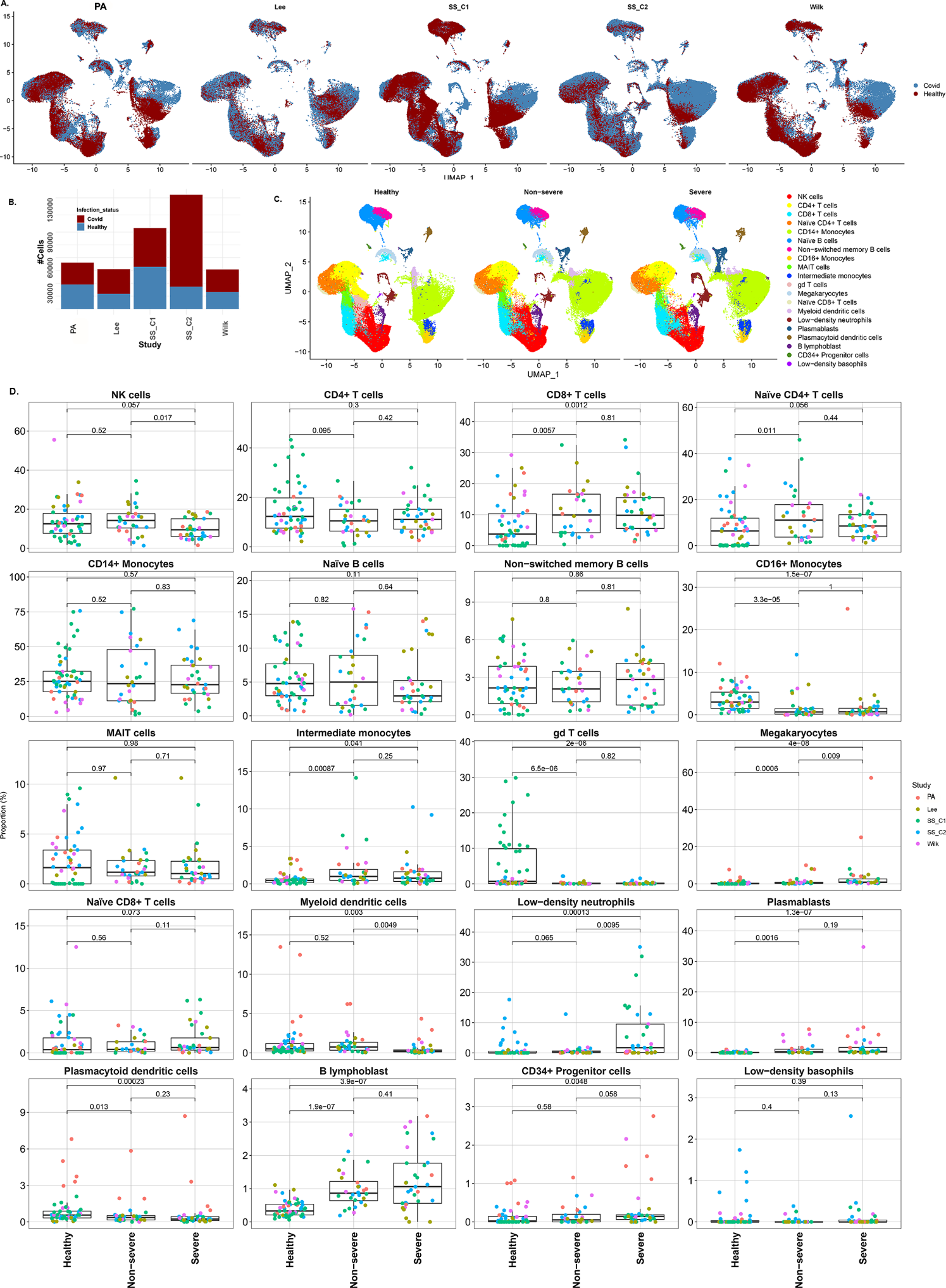


**Supplementary Figure 1. Integrated sc-RNAseq data**: A. This figure summarizes the cell contribution from each of the studies to the integrated dataset. Blue, indicates cells from healthy subjects within the study, while, red indicates cells from COVID-19 subsects within the study. B. The cell contribution from each of the four studies. C. The UMAP embedding of the integrated dataset colored by the 20 distinct cell types across cells from healthy, severe and non-severe subjects. D Boxplots show the proportions of each cell type in each sample colored by study of origin. The x axes correspond to the severity status of each donor. Shown are exact two-sided P values by the Wilcoxon rank-sum test.


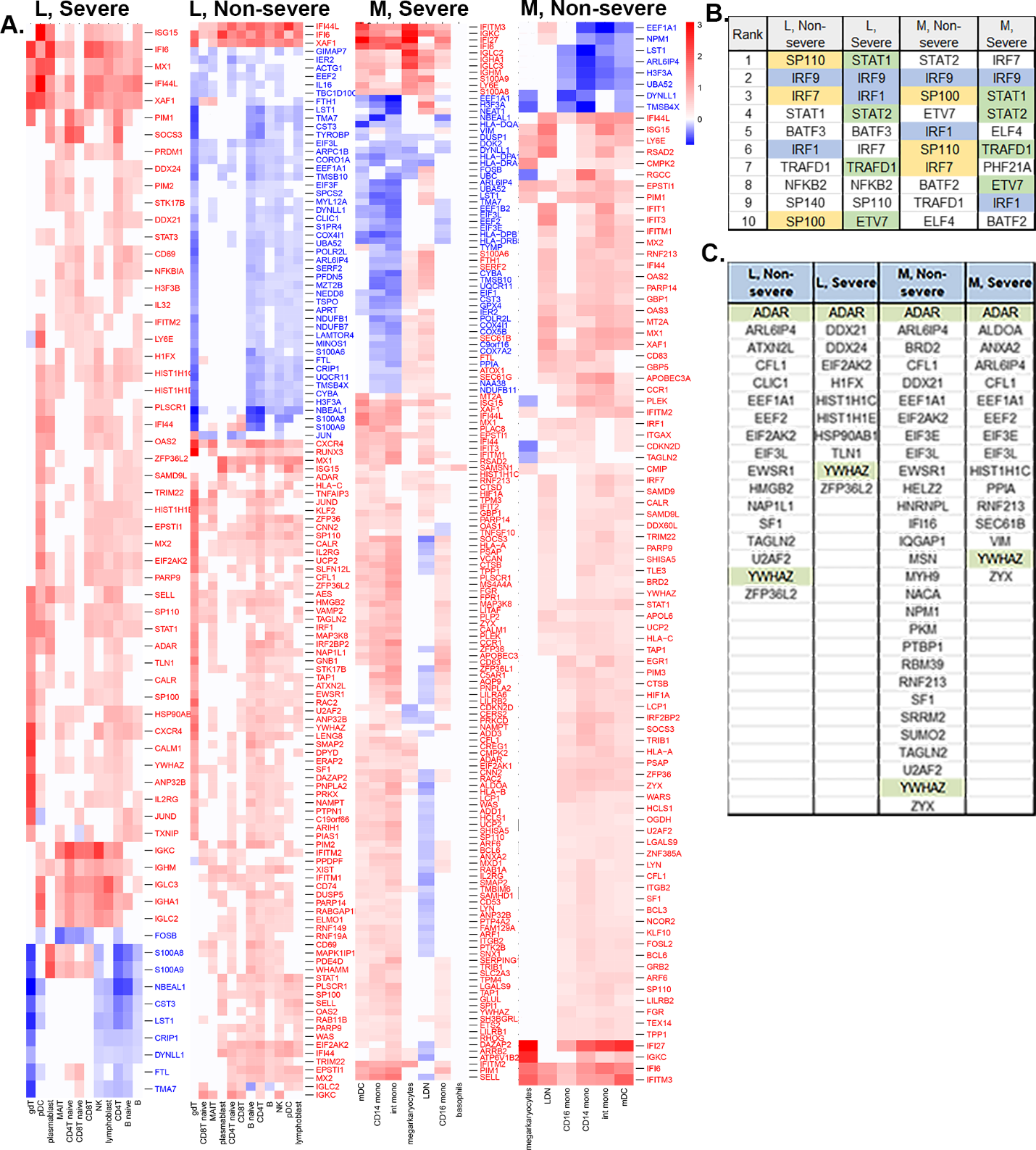


**Supplementary Figure 2. Consensus gene signatures**. A. Heatmap of fold changes for differentially expressed genes identified within the consensus gene set in both severe and non-severe disease (with respect to healthy cells). Shown here for both lymphoid and myeloid cell types. B. Top 10 highly ranked TFs enriched among consensus DEGs. The top 10 TFs were identified using the Top Rank metric available through ChEA3 for consensus DEGs from both lineages across severities are presented here. Common TFs across all four conditions are shown in blue; TFs enriched across cell types in non-severe disease are shown in yellow; and TFs enriched across cell types in severe disease are shown in green. C. The list of RNA binding proteins (RBPs) which were as consensus (expressed in atleast 50% of the celltypess for each lineage) ADAR and YWHAZ are ubiquitously expressed across severities and cell lineages.


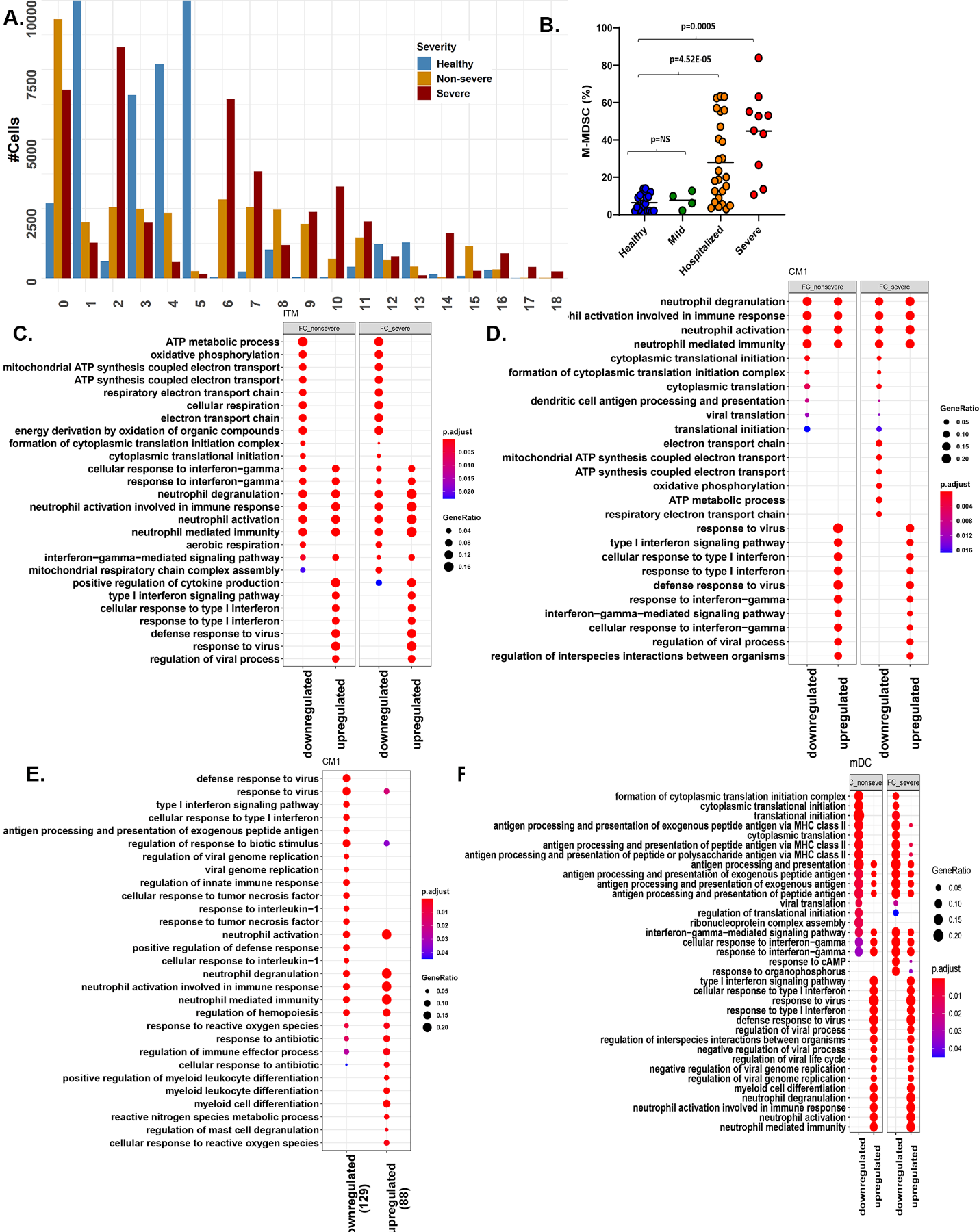


**Supplementary Figure 3. Monocytes and mDC.** A. Cell abundance signatures across severities for the subset monocytes across the 19 clusters identified. B. Immunophenotyping within an independent patient cohort reveals a significant increase in the M-MDSC population within severe (and hospitalized) patients. C and D Enrichment (GO ontology biological process) enrichment of DEGs identified in severe and non-severe cells w.r.t. healthy within subsets ITM and CM1 respectively. E. Enrichment of DEGs identified within the CM1 subset comparing cells from severe to non-severe, identified a prominent suppression of IFN-I response within CM1. F. The enrichment of DEGs mDCs comparing cells from severe to non-severe, indicated a strong IFN response within this subset.


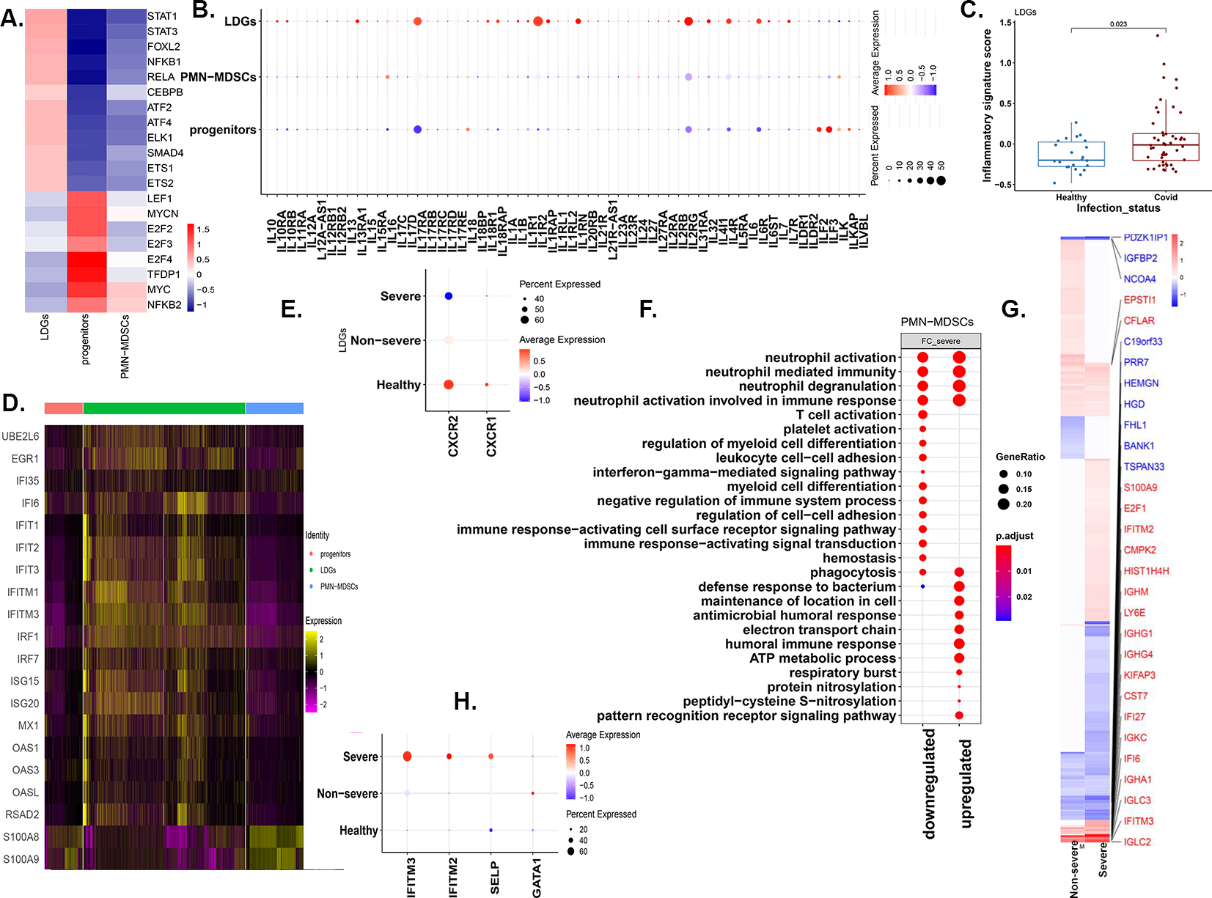


**Supplementary Figure 4. Low-density neutrophils and megakaryocytes.** A. DoRoThEA enrichment identifies increased transcription factor activity of early genes including LEF1, MYC within progenitors while emphasizing increased activity for IFN genes such as STATs and IRFs within LDGs B. A dotplot of average expression for all interleukins identified within LDNs, shows an increased expression within LDGs, alluding to the proinflammatory, interferon responding LDGs C. Inflammatory module score computed using the with Seurat, identifies a significant difference between healthy and COVID-19. D. A heatmap of expression of ISGs and genes activated in response to IFN-I identified within LDNs shows a significant regulation within the cells from LDGs. E. A dotplot highlight the reduced average expression of two proteins, CXCR2 and CXCR1 in severe and non-severe disease among cells annotated as LDGs. F. Enrichment of DEGs identified for PMN-MDSCs, comparing in severe and non-severe disease compared to healthy. G. Heatmap of the fold changes for DEGs identified by comparing severe cells to healthy cells and non-severe cells to healthy within the MK compartment. The top 30 DEGs are explicitly identified. H. Dotplot highlights the reduced expression of GATA1 in MKs, within severe disease as well an increased expression of genes such as SELP within severe disease.


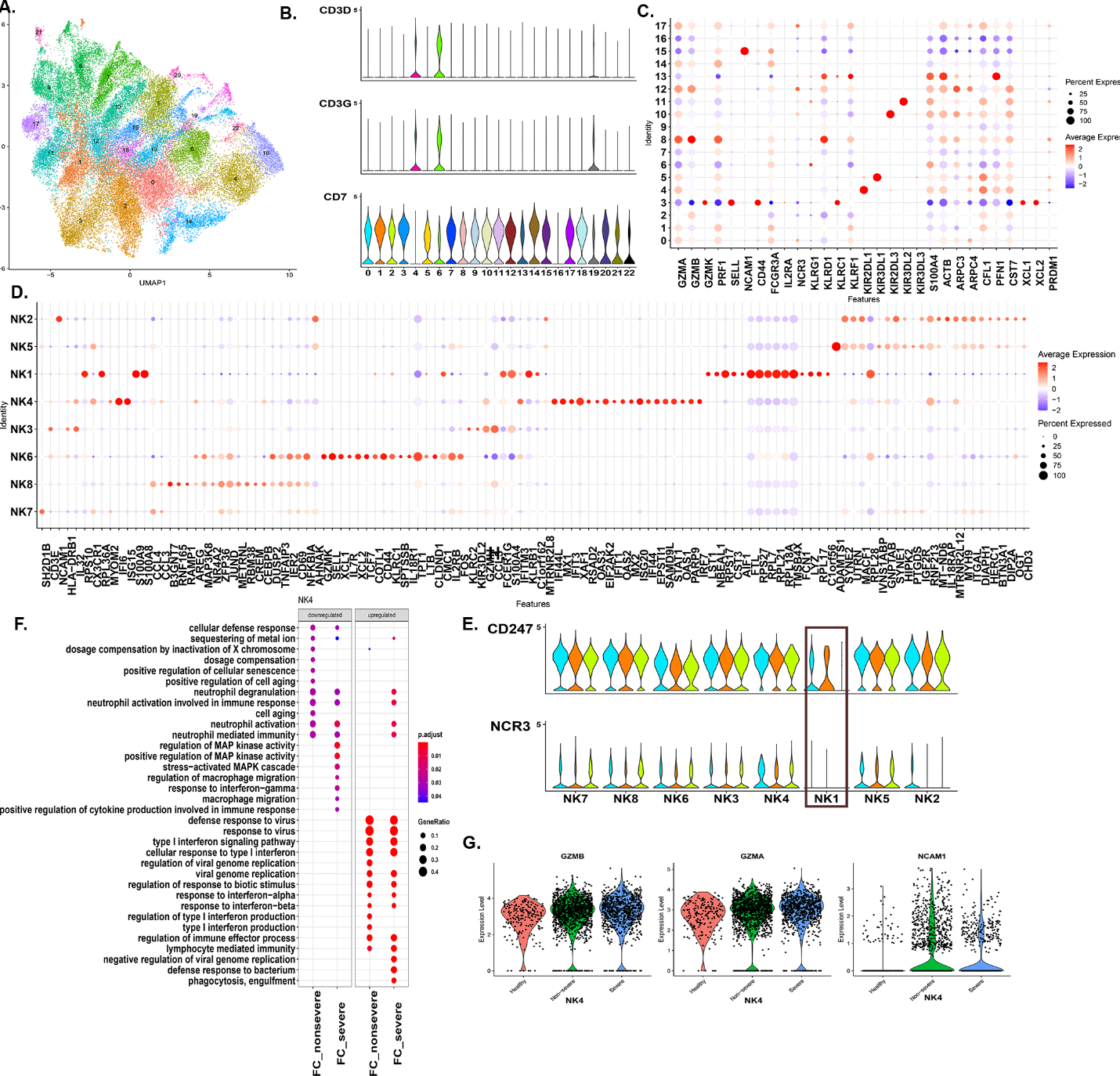


**Supplementary Figure 5. Natural killer cells**. A. The UMAP embedding of 23 clusters of NK cells, subset and re-clustered from the original parent Seurat object. B. Violin plots capturing expression of major markers used to filter out NK-T cells including CD7, CD3D and CD3G. C. A dotplot indicating average expression of specific genes used to group the 18 clusters into NK cell subsets including genes such as the lytic granules such as granzymes (GZMB/K/A), PRF1; inhibitory KIRs; negative regulators including PFN1 and CST7; TFs such as PRDM1 and cytoskeletal proteins including ACTB, ARPC3/4. D. The top 20 cluster markers identified for each NK subset (NK1-NK7). E. The expression of CD247 and NCR3 are marked lower within the NK1 subset highlighting a reduced cytotoxic potential of this subset. F. An enrichment of upregulated and downregulated genes identified in samples from both severe and non-severe compared to healthy are provided in this side-by-side dot plot and highlight the potent upregulation of IFN-I responses in COVID-19. G. Increased expression of granzymes such as GZMB and GZMA and CD56(NCAM1) is seen within the NK4 clusters.


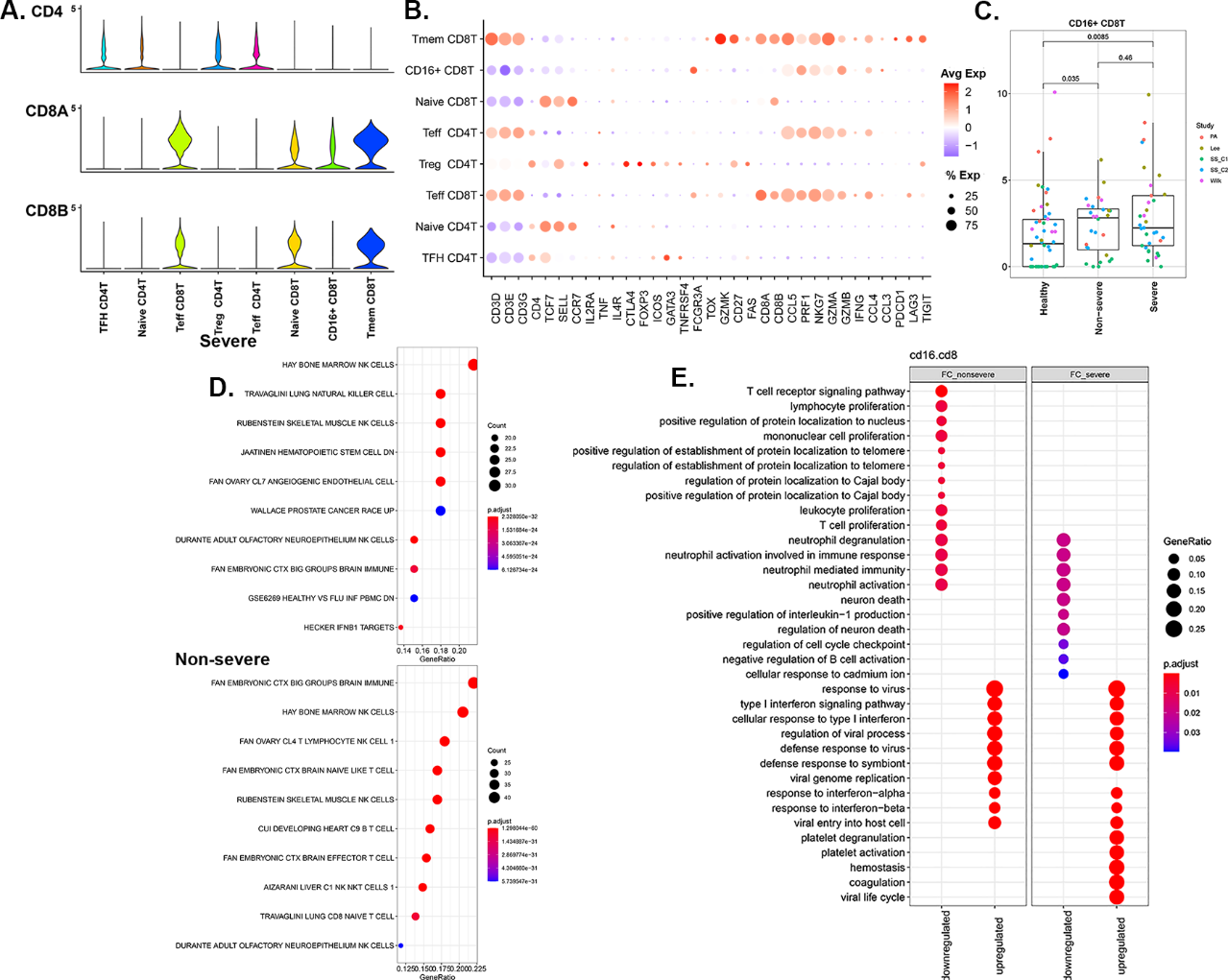


**Supplementary Figure 6. CD4T/8T milieu.** A. Confirms the expression of CD4 and CD8 (A/B) within each of the subsets identified as CD4 and CD8T cells. B. The major markers which define each of the subtypes identified in figure A above, and their average expression is represented within this dotplot. C. A boxplot showing a significant increase (p<0.05) in the proportion of Cd16+ CD8T cells within COVID-19. D. MsigDB enrichment of DEGs identified within CD16+ CD8T cells in both severe and non-severe (compared to healthy) E. A comparative heatmap showing the functional enrichment differences in DEGs identified in severe and non-severe disease (compared to healthy), within the CD16+ CD8T subset


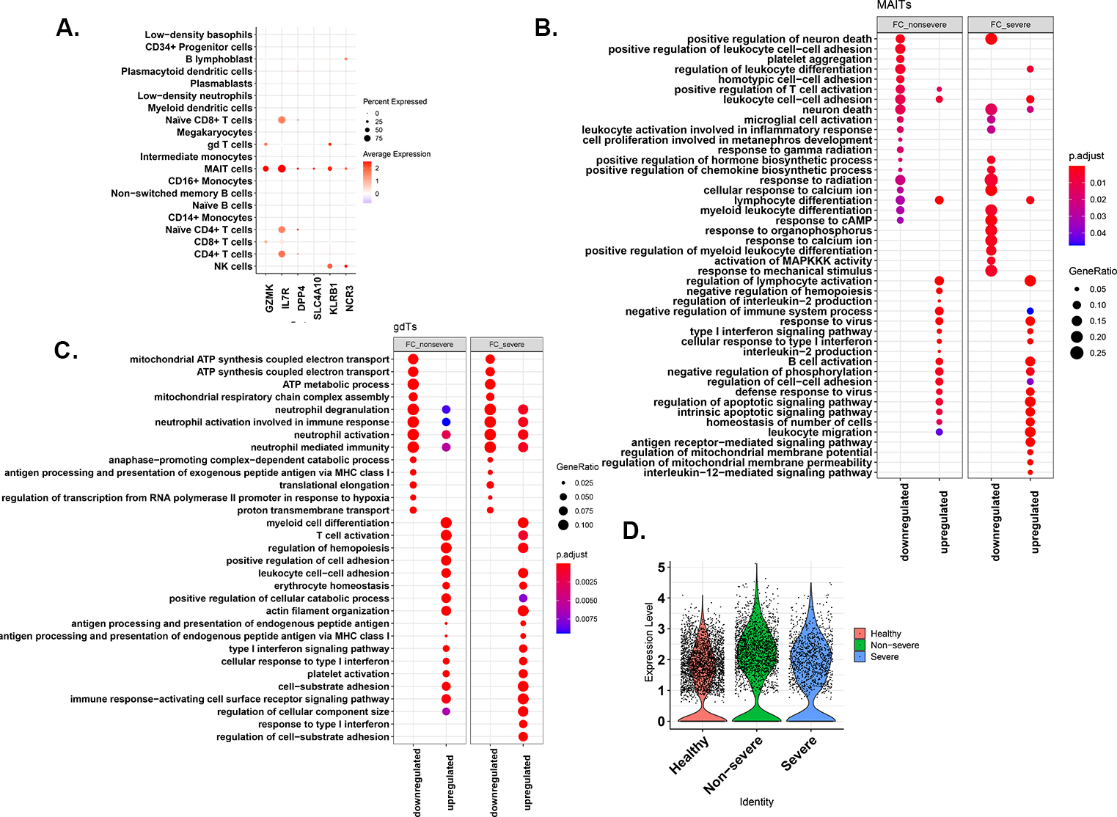


**Supplementary Figure 7. MAITs and gdTs.** A. The dotplot highlights the expression of MAIT specific markers including DPP4, and KLRB1 exclusively within the MAIT clusters of the original parent Seurat object. B. A side-by-side dotplot of enrichment identified for both upregulated and downregulated genes of the MAIT subset within severe and non-severe COVID-19 samples C. A side-by-side dotplot of enrichment identified for both upregulated and downregulated genes of the gdT subset within severe and non-severe COVID-19 samples. D. Violin plot emphasizes the expression level of CD69, an activation marker, increased in MAIT cells from severe and more so, non-severe samples.


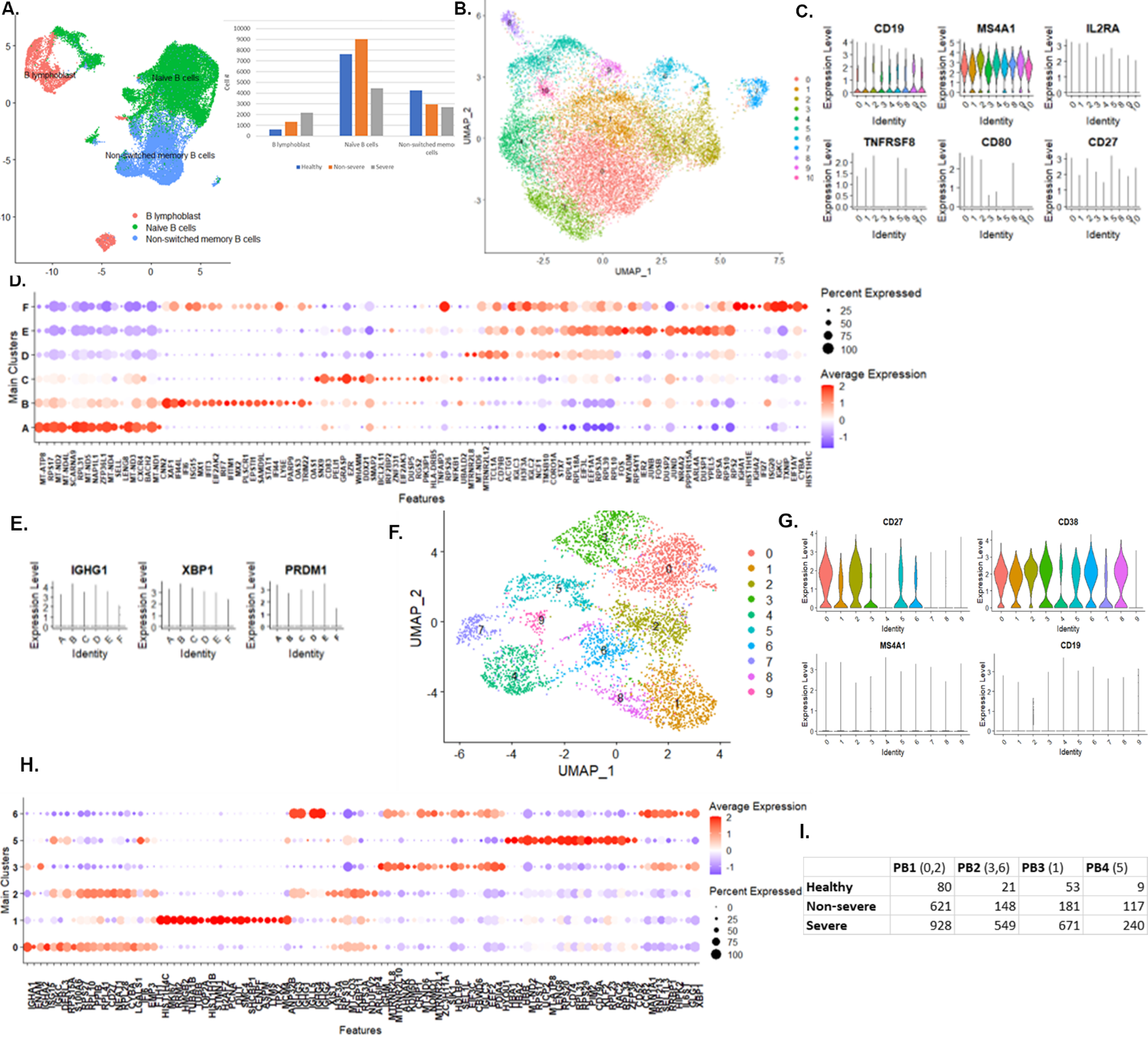


**Supplementary Figure 8. Naïve B-cells and Plasmablasts.** A. UMAP with non-antibody secreting B cells subset from parent Seurat object (For antibody secreting cells, see plasmablast section). Cell type annotations (B lymphoblast, naïve B cells, non-switched memory B cells) from the parent Seurat object are shown. Inset bar graphs shows number of healthy (blue), non-severe (orange), and severe (grey) cells in each B cell category. B. UMAP with subsampled and reclustered naïve B cells with clusters 0-10, prior to excluding non-B cell clusters (See Methods). C. Violin plot verifying high CD19, MS4A1 (CD20) expression with no/low expression of several other markers more indicative of non-naïve B cell fate. D. Dotplot with expression of top 15 cluster markers from each of six final groups A-F. E. Violin plot verifying that naive B cell clusters do not express markers more consistent with plasmablast lineages. F. Subsampling of plasmablasts and sub-clustering of parent Seurat object resulted in ten clusters. G. Six clusters (0,1,2,3,5,6) had expression patterns of cell surface markers that were most consistent with plasmablasts (CD19-, MS4A1- , CD27+, CD38+) and were retained for further analysis. H. Dot plot showing expression of top 20 cluster markers in each of the six retained clusters. Based on expression, six clusters were grouped into four subsets (PB1-4). Clusters 0,2 comprised PB1; clusters 3,6 comprised PB2; and cluster 1 and 5 made up PB3, and PB4 respectively. I. Table shows number of healthy, non-severe, and severe cells in each PB subset. Since healthy cells were only than 5% of all cells, further analyses carried out only on non-severe and severe cells.


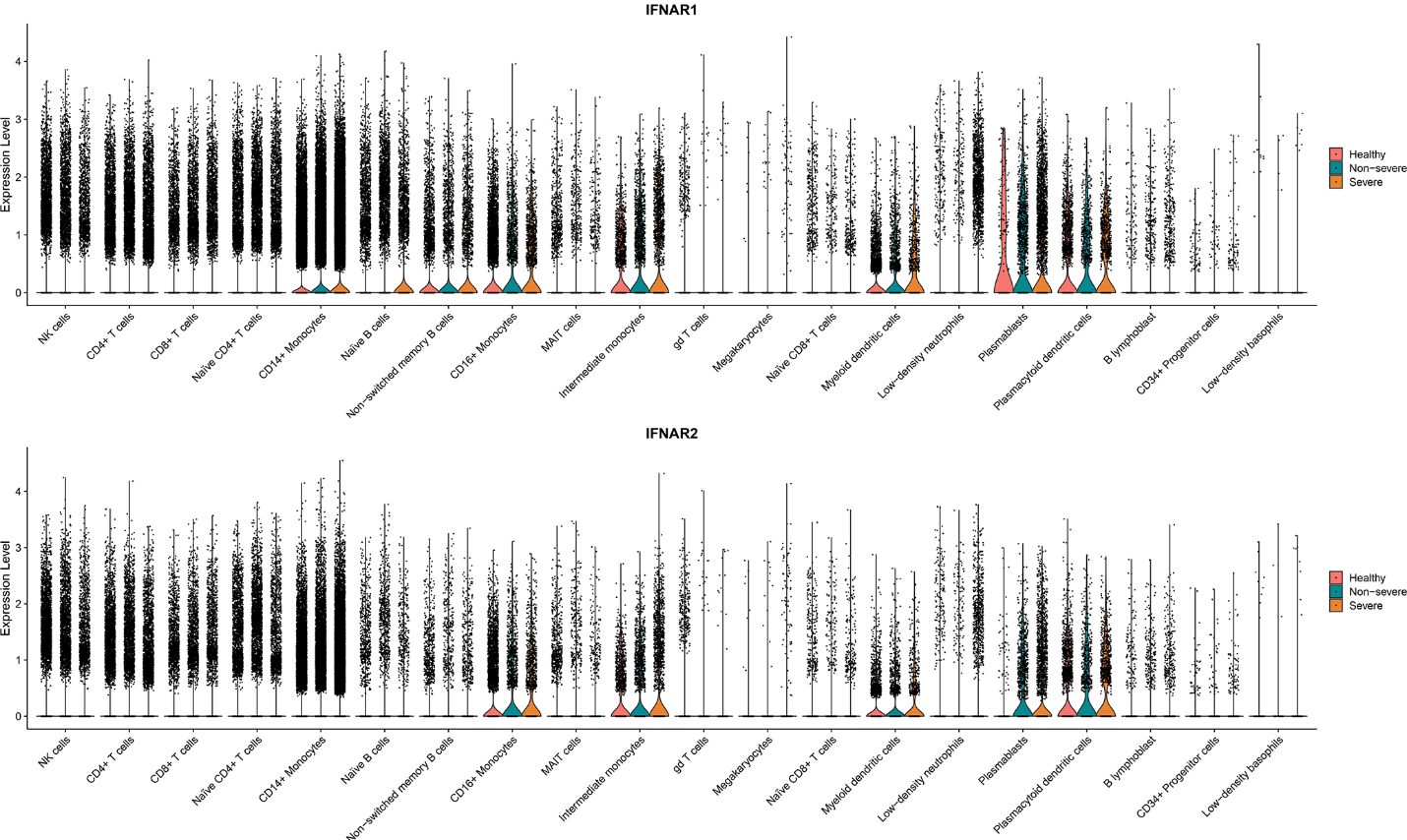


**Supplementary Figure 9. Expression of IFNAR1 and IFNAR2**; two major surface receptors for IFN-I/III identified across the twenty cellular compartments for cells from healthy, severe and non-severe within the integrated dataset.
